# Communication about diagnosis, prognosis, and prevention in the memory clinic: perspectives of European memory clinic professionals

**DOI:** 10.1186/s13195-023-01276-9

**Published:** 2023-08-05

**Authors:** Heleen M. A. Hendriksen, Aniek M. van Gils, Argonde C. van Harten, Tobias Hartmann, Francesca Mangialasche, Anita Kamondi, Miia Kivipelto, Hanneke F. M. Rhodius-Meester, Ellen M. A. Smets, Wiesje M. van der Flier, Leonie N. C. Visser

**Affiliations:** 1 Alzheimer Center Amsterdam, Neurology, Vrije Universiteit Amsterdam, Amsterdam UMC Location VUmc, Amsterdam, The Netherlands; 2 Amsterdam Neuroscience, Neurodegeneration, Amsterdam, The Netherlands; 3 Experimental Neurology, Saarland University, 66424 Homburg, Germany; 4 Deutsches Institut Für DemenzPrävention, Saarland University, 66424 Homburg, Germany; 5 Division of Clinical Geriatrics, Center for Alzheimer Research, Department of Neurobiology, Care Sciences and Society, Karolinska Institutet, Stockholm, Sweden; 6 Karolinska University Hospital, Medical Unit Aging, Theme Inflammation and Aging, Stockholm, Sweden; 7 Department of Neurology, Neurology and Neurosurgery, National Institute of Mental Health, Budapest, Hungary; 8 Department of Neurology, Semmelweis University, Budapest, Hungary; 9 Ageing and Epidemiology (AGE) Research Unit, School of Public Health, Imperial College London, London, UK; 10 Institute of Public Health and Clinical Nutrition, University of Eastern Finland, Helsinki, Finland; 11 Department of Geriatric Medicine, The Memory Clinic, Oslo University Hospital, Oslo, Norway; 12 Internal Medicine, Geriatric Medicine Section, Amsterdam Cardiovascular Sciences Institute, Amsterdam UMC Location VUmc, Amsterdam, The Netherlands; 13 Medical Psychology, Amsterdam UMC Location AMC, University of Amsterdam, Amsterdam, The Netherlands; 14 Amsterdam Public Health, Quality of Care, Personalized Medicine, , Amsterdam, The Netherlands; 15 Epidemiology and Biostatistics, Vrije Universiteit Amsterdam, Amsterdam UMC, Amsterdam, The Netherlands

**Keywords:** Alzheimer’s disease, Dementia, Mild cognitive impairment, Subjective cognitive decline, Clinician-patient communication, Diagnostic workup, Memory clinic, Personalized medicine

## Abstract

**Background:**

The paradigm shift towards earlier Alzheimer’s disease (AD) stages and personalized medicine creates new challenges for clinician-patient communication. We conducted a survey among European memory clinic professionals to identify opinions on communication about (etiological) diagnosis, prognosis, and prevention, and inventory needs for augmenting communication skills.

**Methods:**

Memory clinic professionals (*N* = 160) from 21 European countries completed our online survey (59% female, 14** ± **10 years' experience, 73% working in an academic hospital). We inventoried (1) opinions on communication about (etiological) diagnosis, prognosis, and prevention using 11 statements; (2) current communication practices in response to five hypothetical cases (AD dementia, mild cognitive impairment (MCI), subjective cognitive decline (SCD), with ( +) or without ( −) abnormal AD biomarkers); and (3) needs for communication support regarding ten listed communication skills.

**Results:**

The majority of professionals agreed that communication on diagnosis, prognosis, and prevention should be personalized to the individual patient. In response to the hypothetical patient cases, disease stage influenced the inclination to communicate an etiological AD diagnosis: 97% would explicitly mention the presence of AD to the patient with AD dementia, 68% would do so in MCI + , and 29% in SCD + . Furthermore, 58% would explicitly rule out AD in case of MCI − when talking to patients, and 69% in case of SCD − . Almost all professionals (79–99%) indicated discussing prognosis and prevention with all patients, of which a substantial part (48–86%) would personalize their communication to patients’ diagnostic test results (39–68%) or patients’ anamnestic information (33–82%). The majority of clinicians (79%) would like to use online tools, training, or both to support them in communicating with patients. Topics for which professionals desired support most were: stimulating patients’ understanding of information, and communicating uncertainty, dementia risk, remotely/online, and with patients not (fluently) speaking the language of the country of residence.

**Conclusions:**

In a survey of European memory clinic professionals, we found a strong positive attitude towards communication with patients about (etiological) diagnosis, prognosis, and prevention, and personalization of communication to characteristics and needs of individual patients. In addition, professionals expressed a need for supporting tools and skills training to further improve their communication with patients.

**Supplementary Information:**

The online version contains supplementary material available at 10.1186/s13195-023-01276-9.

## Introduction

Alzheimer’s disease (AD) develops over a prolonged period, including pre-dementia (i.e., preclinical and prodromal) and dementia stages [1]. We know that AD-related pathological changes start 20–30 years before the onset of dementia [2]. Therefore, research has shifted focus to the earlier stages of the disease. These stages provide a window of opportunity for clinical trials and preventive efforts to stop the disease before the onset of dementia [3, 4]. This shifting focus is accompanied by increasing numbers of patients in pre-dementia stages visiting the memory clinic [5, 6].

Many patients visit a memory clinic with a strong need for information about their current disease or health status and the consequences for their daily life now and in the future [7–11]. Biomarkers enable the detection of AD-related pathological changes already in the pre-dementia stages [12] and inform the likelihood of underlying AD as the cause of the patient’s complaints and the patient’s risk of dementia [13]. Nonetheless, communicating about AD biomarker results and dementia risk is challenging [14]. It can both be difficult for the clinician to deliver the message and for the patient to understand it.

There is considerable variation in information needs among patients, for example in when during the diagnostic trajectory a certain topic should be discussed [9]. Individual patients might weigh the pros and cons of receiving information about AD biomarker results, dementia risk, and prevention differently, depending on their preferences and personal situation [15, 16]. Clinician-patient communication personalized to patients’ characteristics, preferences, and needs is a prerequisite for personalized medicine, which could lead to improved quality of life, information recall, reassurance, need fulfillment, and health and disease-related behavior [14, 17, 18]. With disease-modifying therapies (DMTs) and prevention trials showing the first promising results [4, 19, 20], it is even more essential to shift towards a personalized medicine approach, including personalized clinician-patient communication.

It is currently unclear how memory clinic professionals prefer to communicate, whether they personalize their communication, and on what grounds. Such insight is necessary to inform the development of (online) tools and/or skills training that could support professionals in optimizing their communication practices. Therefore, we aimed to survey the opinions of European memory clinic professionals on communicating about (etiological) diagnosis, prognosis, and prevention with patients and their care partners and to which extent they personalize their communication. In addition, we assessed the need for communication tools or training to support professionals in their communication.

## Methods

### Design and participants

We conducted a cross-sectional, online survey study among European professionals working in a memory clinic. This study was conducted in the context of the EU-Fingers (www.eufingers.com) and LETHE (www.lethe-project.eu) consortium projects. Between June and November 2021, we invited memory clinic professionals to participate via our national and international networks by email, newsletters, and websites. Additionally, we recruited memory clinic professionals using snowball sampling (asking participants to forward our invitation to others). An anonymous link to the online survey was included in the invitation. When participants clicked on the link in the invitation, they landed on a page that provided study information. Participants provided digital informed consent before the survey started. Memory clinic professionals, e.g., physicians, nurses, psychologists, working at European memory clinics, i.e., who have clinical experience regarding the diagnostic workup for dementia in a hospital setting, were eligible to participate. Respondents who reported not having any contact with patients in the context of a diagnostic workup for dementia were excluded. From 218 respondents that initially provided informed consent and fulfilled inclusion criteria, 160 respondents completed the survey (73% completion rate). The study was approved by the ethics committee of the Amsterdam UMC.

### Survey

We used Survalyzer software (https://www.survalyzer.com) [21] to collect data online. Since we collected data across Europe, we conducted the survey in English. The survey was piloted among 15 memory clinic professionals from six countries in order to pilot the survey software, allow optimization of the survey in terms of wording and completion time, and check whether all questions made sense from the perspective of memory clinic professionals working in different health care systems. The survey consisted of four parts: (1) characteristics, (2) statements, (3) patient cases, and (4) needs and preferences for communication support (see Survey, Additional file 1).

First, we collected professionals’ age, gender, and profession and asked about the organization and diagnostic procedures of their memory clinic. Additionally, we used an adapted version of the Control Preferences Scale (CPS) [22] to assess their preferred role in decision-making about AD-biomarker testing (one item). We used items from the Physician’s Reaction to Uncertainty Scale (PRUS) [23] to assess how they cope with uncertainty. We selected 12 items from three subscales; anxiety due to uncertainty, concern about bad outcomes, and reluctance to disclose uncertainty to patients. Answers were given on a 6-point Likert scale ranging from “strongly disagree” to “strongly agree”; a higher score means a lower ability to cope with uncertainty (range 12–72).

Second, we explored professionals’ opinions and preferences regarding communication about (etiological) diagnosis, dementia risk and prevention, and patient involvement and support, using 11 study-specific statements on a 5-point Likert scale ranging from “strongly disagree” to “strongly agree.” Next, we asked who should initiate the communication on prevention, i.e., “In your view, who’s most responsible for discussing prevention with regard to cognitive decline/brain health with patients?” (five options; scores weighted with the inverse of the rank: (1) the general practitioner/primary care physician, (2) the referring medical doctor, (3) the memory clinic nurse, (4) the memory clinic treating medical doctor, or (5) public health organizations (e.g., via government campaigns)).

Third, we asked professionals about their preferred communication practice by providing them with five patient cases: 1: dementia due to AD with abnormal AD biomarkers (AD dementia), 2: MCI with normal AD biomarkers (MCI −), 3: MCI with abnormal AD biomarkers (MCI +), 4: SCD with normal AD biomarkers (SCD −), and 5: SCD with abnormal AD biomarkers (SCD +). For each case, we asked professionals how they would communicate about (etiological) diagnosis, prognosis, and prevention to the hypothetical patient during the disclosure consultation by using closed-ended questions. Example questions included: “Would you communicate dementia/Mild Cognitive Impairment (MCI)/Subjective Cognitive Decline (SCD) as the patient’s syndrome diagnosis/diagnostic label?,” “Would you communicate the biomarker results to this patient?,” or “Would you communicate prognosis in terms of dementia risk to this patient?.”

Fourth, we provided professionals with ten communication skills based on skills deemed important for healthcare professionals in general, as well as skills that are specific to the context of the memory clinic (based on previous research and/or input on our pilot survey) [14, 24, 25]. We asked to what extent they would prefer communication support using a 5-point Likert scale. In addition, we asked if they would like to use communication tools and/or communication skills training to support their communication with patients (5 response options: 1: Yes, communication tools, 2: Yes, skills training, 3: Yes, both, 4: No, 5: I don’t know).

### Statistics

We used descriptive statistics to report characteristics and survey responses. Frequencies and percentages were calculated. Additionally, we used Kruskall-Wallis tests, Mann–Whitney U tests, and Spearman’s rho analyses when appropriate, to assess correlations between professionals’ characteristics (e.g., age, years of experience) and the mean need for support. SPSS-statistics software version 26 was used for the analyses. *P*-values < 0.05 were considered significant.

## Results

### Sample characteristics

**Fig. 1:**
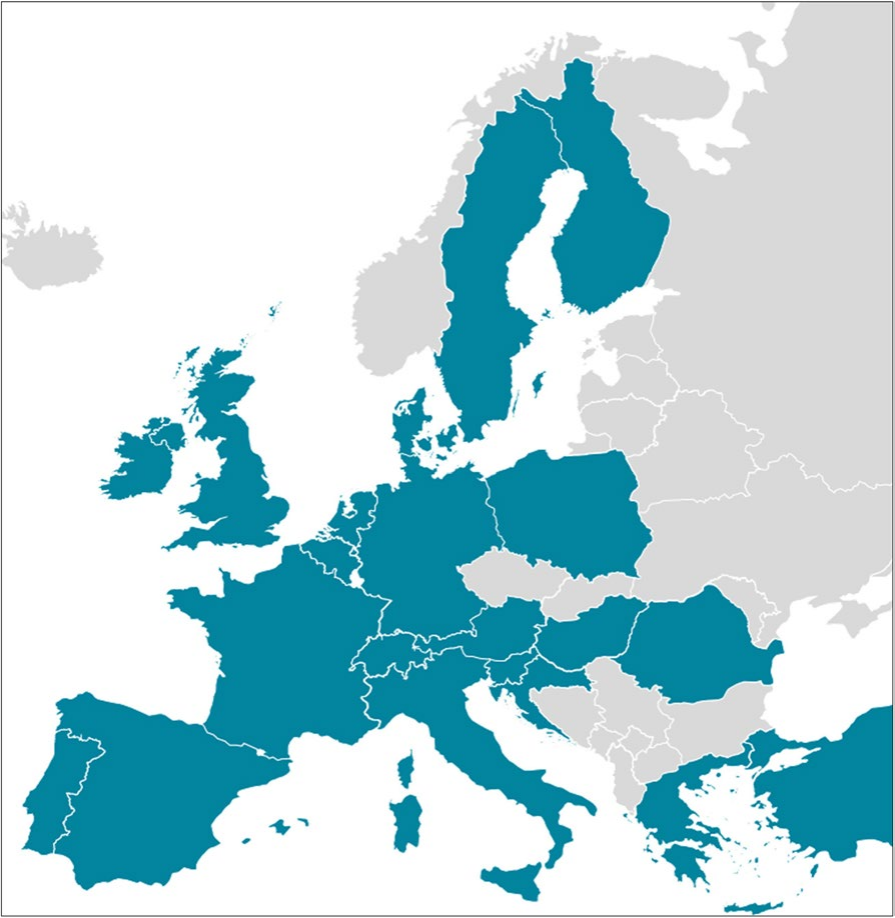
Memory clinic professionals (*N* = 160) from 21 European countries completed the survey. The Netherlands: *n* = 37 (23%); Germany: *n* = 17 (11%); Hungary: *n* = 13 (8%); Spain: *n* = 11 (7%); UK: *n* = 11 (7%); Austria: *n* = 10 (6%); Finland: *n* = 10 (6%); Sweden: *n* = 10 (6%); Switzerland: *n* = 7 (4%); Portugal: *n* = 7 (4%); Italy: *n* = 6 (4%); France: *n* = 6 (4%); Greece: *n* = 4 (3%); Belgium: *n* = 3 (2%); Ireland: *n* = 2 (1%); Croatia: *n* = 1 (1%); Denmark: *n* = 1 (1%); Poland: *n* = 1 (1%); Romania: *n* = 1 (1%); Slovenia: *n* = 1 (1%); Turkey: *n* = 1 (1%)

As visualized in Fig. 1, 160 memory clinic professionals from 21 European countries completed the survey. Sample characteristics are shown in Table 1. Of these professionals, 95 (59%) were female and they were rather evenly distributed across age groups. The memory clinic professionals had an average 14 ± 10 years of experience, most worked in neurology (90, 56%), and in an academic/university hospital (116, 73%). In terms of profession, 137 (86%) were physicians, and 23 (14%) were (specialized) nurses, nurse practitioners, or (neuro)psychologists.

**Table 1:** Sample characteristics

	***n* = 160**
**Female, *n* (%)**	95 (59%)
**Years of experience (mean ± SD)**	14 ± 10
**Age, *n* (%)**	
18–30 years old	19 (12%)
31–40 years old	45 (28%)
41–50 years old	44 (28%)
51–60 years old	27 (17%)
61–70 years old	22 (14%)
71–80 years old	3 (2%)
**Profession, *n* (%)**	
Physician, after completion of specialist training	122 (76%)
Physician, without or currently in specialist training	15 (9%)
Other, e.g., (specialized) nurse, nurse practitioner, or (neuro)psychologist	23 (14%)
**Medical specialty, *n* (%)**	
Neurology	90 (56%)
Internal/Geriatric medicine	36 (23%)
Psychiatry	31 (19%)
Other	3 (2%)
**Type of organization/hospital, *n* (%)**	
Academic/university hospital	116 (73%)
Non-academic teaching hospital	29 (18%)
Other, e.g., non-teaching hospital or mental health service	15 (9%)

Data are presented as *n* (%) or mean ± SD

### Statements regarding communicating diagnosis, prognosis and prevention, and patient involvement and support

**Fig. 2:**
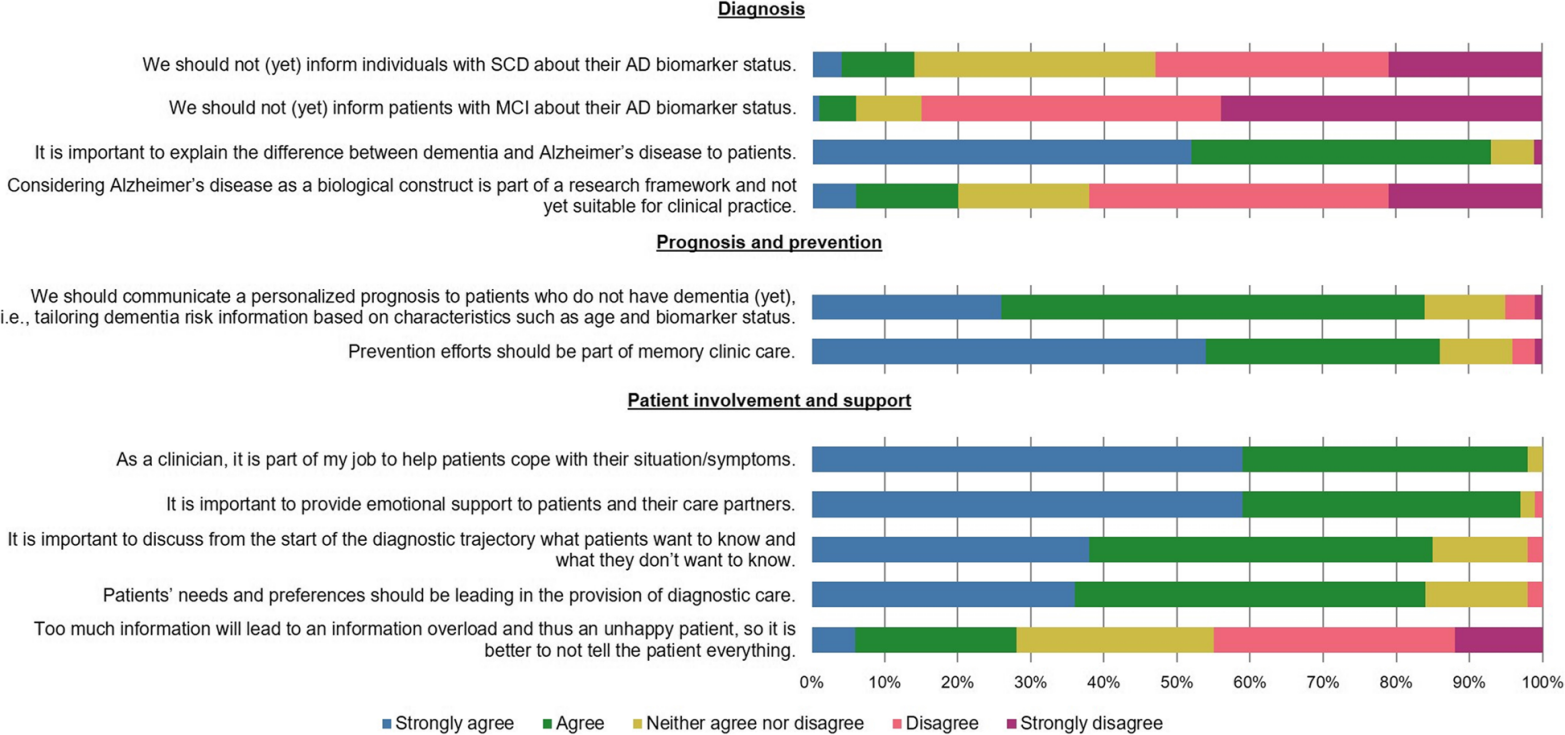
Responses to statements regarding communicating about diagnosis, prognosis and prevention, and patient involvement and support. *Abbreviations:* SCD, subjective cognitive decline; MCI, mild cognitive impairment; AD, Alzheimer’s disease

The responses of professionals to the 11 statements are presented in Fig. 2. The figure is divided into three parts: 1) diagnosis, 2) prognosis and prevention, and 3) patient involvement and support. Regarding diagnosis, a clear majority (93%) (strongly) agreed that it is important to explain the difference between dementia and Alzheimer’s disease to patients. Furthermore, a vast majority (85%) of professionals (strongly) *dis*agreed with the statement that we should not (yet) inform individuals with MCI about their AD biomarker status. For SCD, opinions were heterogeneous. Approximately half of the professionals disagreed (53%), one-third (33%) neither agreed nor disagreed, and 14% agreed.

Regarding prognosis and prevention, a vast majority (84%) of professionals (strongly) agreed that professionals should communicate a personalized prognosis to patients who do not have dementia (yet). A similar proportion of 85% of professionals (strongly) agreed that preventive efforts should be part of memory clinic care. In addition (not included in Fig. 2), 43% indicated that the memory clinic treating medical doctor is primarily responsible for discussing prevention with patients, followed by public health initiatives (e.g., through government campaigns) (25%), the general practitioner/primary care physician (24%), the memory clinic nurse (7%), and the referring medical doctor (1%).

Regarding patient involvement and support, a vast majority (strongly) agreed that it is important to inquire at the start of the diagnostic trajectory what patients do and do not want to know (85%) and that patients’ needs and preferences should be leading in the provision of diagnostic care (84%). Professionals provided diverse reactions regarding the statement that “too much information will lead to an information overload and thus an unhappy patient, so it is better to not tell the patient everything,” where 6% of professionals strongly agreed, 22% agreed, 27% neither agreed nor disagreed, 33% disagreed, and 12% strongly disagreed.

### Patient cases

Subsequently, we provided professionals with five hypothetical patient cases and asked how they would communicate about (etiological) diagnosis, prognosis, and prevention (see Survey, Additional file 1 and Supplementary Tables 1–2, Additional file 2).

#### Diagnosis

Regarding communication of diagnosis, most professionals (66–88%) indicated they would communicate the relevant syndrome diagnosis (i.e., dementia, MCI, or SCD) to the five hypothetical patients (see Supplementary Table 1, Additional file 2). In all five cases, almost all professionals (97–100%) would communicate the AD biomarker test results to the patient, although with different interpretations. In the dementia case, there is consensus about the communication strategy of the AD biomarker results, as 97% would communicate AD as the underlying pathology to the patient, and 92% would explain the difference between dementia and AD.

**Fig. 3:**
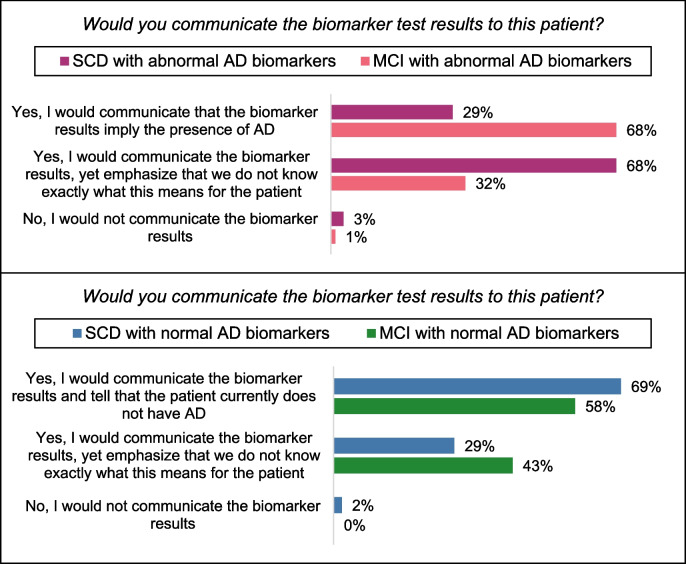
Memory clinic professionals’ answers to: “Would you communicate the biomarker test results to this patient?”* Abbreviations:* SCD, subjective cognitive decline; MCI, mild cognitive impairment; AD, Alzheimer’s disease

Within and between the syndrome diagnoses MCI and SCD, professionals varied in how they would communicate the meaning of AD biomarker test results. If AD biomarkers were *abnormal*, 108 professionals (68%) would communicate AD as the underlying pathology to the patient with *MCI*, while 51 professionals (32%) would communicate to the same patient that it is not yet exactly known what the test results mean for the patient. Conversely, in case of *SCD* with abnormal AD biomarkers, 46 professionals (29%) would communicate the presence of AD pathology, while 109 professionals (68%) would communicate to the same patient that it is not yet known what the test results exactly mean (see Fig. 3, upper figure).

In case of *normal* AD biomarkers, 92 professionals (58%) would communicate that the patient currently does not have AD in case of *MCI*, while 68 professionals (43%) would communicate to the same patient that it is not yet exactly known what the test results mean. In case of *SCD* with normal biomarkers, 110 professionals (69%) would communicate that the patient currently does not have AD pathology, while 47 professionals (29%) would communicate to the same patient that it is not yet exactly known what the test results mean (see Fig. 3, lower figure).

#### Prognosis and prevention

**Fig. 4:**
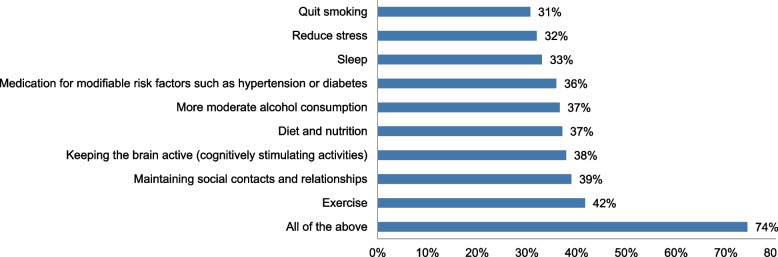
Potential ways to reduce the risk of dementia/cognitive decline that professionals indicate to address

Almost all professionals would discuss prognosis (79–98%) and prevention (90–99%) with all five hypothetical patients. A substantial part would do so according to the patient’s preferences, e.g., “only if the patient or partner prefers to know” (20–36% when discussing prognosis, and 9–25% when discussing prevention). In addition, many professionals indicated personalizing their communication regarding these topics based on patient characteristics (48–65% when discussing prognosis, and 73–86% when discussing prevention; see Additional file 2). These characteristics entail patient’s anamnestic information (33–82%, e.g., what patients have told about lifestyle) and diagnostic test results (39–68%, e.g., cognitive tests, AD biomarker results). Professionals would often address multiple preventive efforts to potentially reduce the risk of dementia/cognitive decline (see Fig. 4).

### Need and preferences for communication support

**Fig. 5:**
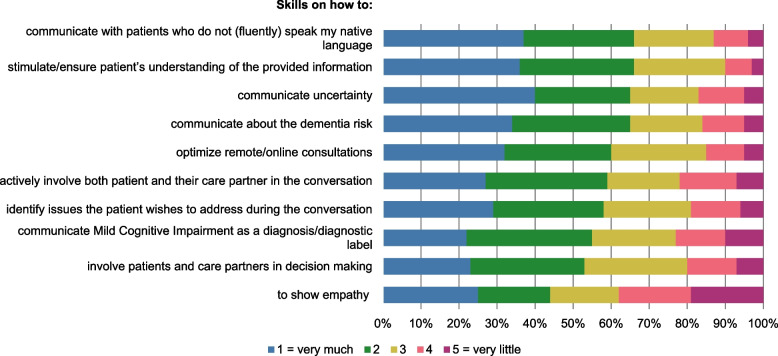
The amount of support memory clinic professionals wish to receive for ten listed communication skills

In the final part of the survey, professionals expressed their interest in support for clinician-patient communication. A majority of professionals (79%) would like to use online communication tools (34%), take part in communication skills training (13%), or both (32%). For 9/10 listed communication skills, more than half of these professionals preferred ‘(very) much’ support (see Fig. 5). They most often indicated a need for support on (1) communicating with patients not (fluently) speaking the language of the country of residence (66%), (2) stimulating/ensuring patient’s understanding of the information provided (66%), (3) communicating uncertainty (65%), (4) communicating about dementia risk (65%), and (5) optimizing remote/online consultations (60%).

Table 2 shows professionals’ history of participation in communication skills training, ability to cope with uncertainty, and preferred role in decision-making. Correlation analysis showed that professionals with a lower self-reported ability to cope with uncertainty expressed a stronger need for support (*r*(140) = 0.309, *p* < 0.001). No other associations were found between professionals’ characteristics, such as age group, sex, years of experience, preferred role in decision-making (CPS), specialty, or type of organization/hospital, and the mean need for communication support.

**Table 2:** Participation in communication skills training, ability to cope with uncertainty, and preferred role in decision-making

	***n* = 160**
**Have you participated in any communication skills training/courses?**	
Yes, both during my education as after completion of my education	40 (25%)
Yes, after completing my education	20 (13%)
Yes, as part of my education	65 (41%)
No	35 (22%)
**How long ago was the most recent communication course that you participated in?** (*n* = 125)	
This month	8 (6%)
This year	21 (17%)
More than a year ago	44 (35%)
More than 5 years ago	36 (29%)
More than 10 years go	16 (13%)
**Ability to cope with uncertainty (PRUS)**	32.5 ± 8.4
**Preferred role in decision-making about AD biomarker testing (CPS)**	
The patient makes the final decision about whether or not to pursue diagnostic testing and which tests to use	10 (7%)
The patient makes the final decision about whether or not to pursue diagnostic testing and which tests to use after seriously considering the clinician’s/my opinion	36 (27%)
The patient and the clinician/I share the responsibility for deciding about whether or not to pursue diagnostic testing and which tests are best for the patient	66 (49%)
The clinician makes/I make the final decision about whether or not to pursue diagnostic testing and which tests to use, but seriously consider the patient’s opinion	20 (15%)
The clinician makes/I make the final decision about whether or not to pursue diagnostic testing and which tests to use	2 (2%)

Data are presented as n (%) or mean ± SD. PRUS: a higher score means a lower self-reported ability to cope with uncertainty (potential range 12–72)

*Abbreviations: PRUS* Physician’s Reaction to Uncertainty Scale, *AD* Alzheimer’s disease, *CPS* Control Preferences Scale

## Discussion

This pan-European survey shows that memory clinic professionals have a strong positive attitude towards optimizing and personalizing their communication with patients about (etiological) diagnosis, prognosis, and prevention. Although most professionals indicated to communicate about these topics in response to our hypothetical patient cases, they differed in how they would explain the meaning of (ab)normal biomarker results to patients with SCD or MCI. In addition, they expressed a clear need for communication support, by means of communication tools and skills training.

Our study extends on former literature on memory clinic professionals’ perspectives regarding patient communication by obtaining a broader view using hypothetical patient cases, focusing on pre-dementia stages of AD, including preclinical AD, actively assessing the influence of AD biomarker status on communication practices, and enquiring about whether communication should be personalized and on what grounds.

In previous (inter)national survey studies, percentages of clinicians indicating to always disclose a diagnosis ranged from 68 to 94%, and discuss prognosis varied from 39 to 77% [24, 26–29]. Of note, these studies were conducted in the context of MCI and dementia, and not of SCD. The terms used by clinicians to discuss diagnosis were heterogeneous, varying from “MCI,” “memory loss,” “possible early dementia,” “emphasizing that there is no AD or dementia,” “possible early AD,” “Alzheimer disease,” “memory disease,” “dementia,” and “degenerative disease.” In our current pan-European sample, 77–88% would communicate an MCI and/or dementia syndrome diagnosis, 97% would communicate an etiological diagnosis of AD to a patient with dementia, and 68% would communicate an etiological diagnosis of AD to a patient with MCI. Regarding discussing prognosis, 79–97% of clinicians in our sample indicated discussing prognosis, and this percentage is even higher when we exclude the SCD patient case (89–97%). In sum, regarding discussing diagnosis, our study shows similar results compared to previous studies, although in our study, a clearer distinction between syndrome and etiological diagnosis was made. Regarding discussing prognosis, our sample notably favors discussing prognosis.

The greater preference to discuss prognosis in our sample when compared to previous research might be due to the rapid diagnostic advancements in the field and the increasing knowledge about AD, as memory clinic professionals have more informative data to convey to patients. AD has shifted from a disease that could only be clinically described and/or diagnosed post-mortem, to a disease in which neuropathological changes can be identified antemortem [30, 31]. These developments have occurred in a time frame of only several decades, with the establishment of the NIA-AA research framework toward a biological definition of AD in 2018 [13]. This also explains the heterogeneity of diagnostic terms used in previously conducted surveys, which makes it difficult to compare results and digest what professionals tell patients when they indicate to discuss diagnosis. In our study, 93% of professionals agreed that it is important to explain the difference between dementia and Alzheimer’s disease, or in other words, between a syndrome diagnosis and an etiological diagnosis. This supports the notion that the clinicians increasingly see Alzheimer’s disease as a biological construct, more equally involving the full range of syndromes across the continuum, including those in preclinical and prodromal stages.

Regarding those pre-dementia stages, clinicians in our study differed in explaining the meaning of abnormal AD biomarkers to patients. In pre-dementia stages, this explanation concerns a prognosis rather than a diagnosis, with inherent uncertainty. For MCI, (individualized) prediction models are available to provide an estimation of the risk to develop dementia, for example by taking into account a patient’s age, sex, and results on cognitive or AD biomarker tests [32, 33]. For SCD, it is known that individuals with abnormal AD biomarkers have an elevated dementia risk on a group level [34–37]. However, it is more difficult to estimate dementia risk on an individual level due to the current lack of longitudinal data with sufficient duration of follow-up and end-points reached, as clinical progression in this stage takes longer. In addition, due to heterogeneity in operationalization of SCD and sufficient follow-up, external validation in multiple SCD cohorts has proven challenging [38]. As a result, variability in opinions and practices among professionals is higher. Nonetheless, an increasing number of papers is emerging showing the predictive value of biomarkers in cognitively normal individuals [35, 36]. The implementation of prediction models in clinical practice (after improvements through longer follow-up), could further aid professionals with what to communicate to patients in pre-dementia stages of AD. Interestingly, we also found considerable variability in communication practices in case of *normal* AD biomarkers, while one would argue that ruling out AD pathology is of high relevance patients. Furthermore, in our sample, 85% (strongly) agreed that it is important to inquire at the start of the diagnostic trajectory what patients do (not) not want to know, and that patients’ needs and preferences should be leading in the provision of diagnostic care (84%). Communication tools that support clinicians in exploring those needs and preferences and managing expectations with regards to diagnostic work-up, could help clinicians in providing the desired care.

While our findings provide insight into professionals’ preferred communication strategies, this preference might not (yet) translate to actual clinical practice. In our study, the majority of professionals would explicitly mention AD to the hypothetical patient with MCI and abnormal AD biomarkers and also provide a personalized prognosis, which aligns with recommendations [39]. However, results from an observational study show that clinicians were quite tentative in addressing the cause of MCI patients’ symptoms, even when biomarker information was available [40]. Furthermore, few clinicians provided a personalized prognosis, meaning that demographic or clinical characteristics, or (ab)normal biomarker results were not considered. In addition, active patient involvement in consultations seemed limited whereby clinicians seldom invited patients to express their questions, needs, and preferences [40]. These findings illustrate the communication challenges that memory clinic professionals encounter and highlight the importance of communication support by means of digital tools or skills training to assist professionals in these challenges.

In other medical contexts, such as oncology, it was found that communication skills do not reliably improve with experience alone [41]. Meanwhile, communication skills training for healthcare professionals appeared effective in improving communication behavior, such as personalizing communication according to patients’ emotions and needs [42]. In our study, memory clinic professionals expressed a (strong) need for support with respect to a variety of communication skills, particularly regarding stimulating patients’ understanding of information, communicating with patients not (fluently) speaking their native language, communicating dementia risk, communicating uncertainty, and online/remote communication. Interestingly, professionals with relatively greater difficulties coping with uncertainty expressed a stronger need for communication support. A Dutch survey study showed that memory clinic professionals who reported greater difficulties coping with uncertainty also preferred a bigger say in medical decision-making than the patient [24]. It thus seems that the professional’s ability to cope with uncertainty influences their interaction with patients. The available evidence on professionals' uncertainty tolerance was systematically reviewed, suggesting among other things that lower uncertainty tolerance is associated with higher psychological distress [43]. It might be that the experience of psychological distress during clinical encounters, impedes cognitive processing and influences the behavior of the health care professional, thereby affecting the interaction with the patient. Yet, the presence of measurement variability across studies included in the review made it difficult to draw definitive conclusions about factors that contribute to, or result from, uncertainty tolerance [43]. Despite the need for additional research on this topic, communication tools or skills training could already try to empower professionals in effectively managing and conveying uncertainty, thereby benefiting both themselves as well as patients [44]. Overall, our findings support the notion that many professionals, independent of their experience level, gender, or age, have a positive attitude toward optimizing their communication skills and personalized communication with patients in practice. This is important, as such a widely supported need across memory clinic professionals enables a process of change [45].

Among the strengths of our study is the international perspective that fosters the generalizability of results. Furthermore, we used hypothetical patient cases to increase the relevance for clinical practice. The relevance and applicability of the survey are supported by the high completion rate of above 70%. However, a limitation of our study is a potential response bias, in the sense that we have included professionals who already had a greater interest in the topic of communication, i.e., our findings could overestimate professionals’ positive attitude towards optimizing and personalizing their communication with patients. Furthermore, although many European countries are represented in the survey, some countries were represented by only one or a few clinicians. Also, 160 survey respondents is a fraction of European memory clinic professionals overall, however, in comparison to previous European survey studies with less survey respondents, 160 is a substantial number [27, 28]. In addition, three-fourths of the memory clinic professionals in our study worked in an academic setting. Hence, our results may not be generalizable to memory clinics in a non-academic setting, where biomarker assessment is less often performed. Indeed, a Dutch qualitative interview study with five general practitioners and six specialists (geriatricians and neurologists) working in a community hospital showed less favorable attitudes toward AD diagnostics in SCD or MCI [46]. Further research could actively target professionals in a non-academic setting, to see how our current findings translate.

The current study can serve as starting point for the discussion on optimizing communication on (etiological) diagnosis, prognosis, and prevention in memory clinics. Enhancing communication practices might be even more important in the future when disease-modifying therapies (DMTs) become available, as it requires communication on applicability to the individual patient [47], expected results of the therapy, and chance of side effects. In addition, our findings support the further development of tools and skill training. To date, there are several (digital) tools and guidelines available that support memory clinic professionals in communicating diagnosis and prognosis across the disease continuum, including individualized prediction models, and in engaging patients and their families in the diagnostic trajectory [14, 48–51]. Although clinicians report a positive attitude towards digital tools, including communication aids, for memory clinics these tools are not yet widely implemented in clinical practice [52]. In oncology, a lack of co-creation and attention to effectiveness with robust outcome measurements have been found to hamper implementation into clinical practice [53]. Thus, we recommend future studies to test effectiveness and conduct implementation studies to (further) develop evidence-based tools and communication skills training, together with stakeholders, in order to actually make the transition to clinical practice. The positive attitudes of professionals, as reported in our survey, show a strong support base for this future work.

## Conclusion

In a pan-European survey of memory clinic professionals, we found a strong positive attitude towards optimizing and personalizing communication with patients about (etiological) diagnosis, prognosis, and prevention. The professionals expressed a need for supporting communication tools and skills training to further improve their communication with individual patients in memory clinic practice. These findings can inform the (further) development of such tools and communication skills training programs, and aid the implementation process.

### Supplementary Information

**Additional file 1.** Survey. The perspectives of memory clinic clinicians on (communicating about) early diagnosis of Alzheimer’s disease, dementia risk and prevention: a EU-FINGERS & LETHE survey.

**Additional file 2: Supplementary Table 1.** Patient cases: communicating about diagnosis and diagnostic tests. **Supplementary Table 2.** Patient cases: communicating about prognosis and prevention.

## Data Availability

The datasets used during the current study are available from the corresponding author on reasonable request.

## Abbreviations

AD: Alzheimer’s disease
CPS: Control Preferences Scale
DMTs: Disease-modifying therapies
MCI: Mild cognitive impairment
PRUS: Physician’s Reaction to Uncertainty Scale
SCD: Subjective cognitive decline

## References

[1] Scheltens P, De Strooper B, Kivipelto M, Holstege H, Chetelat G, Teunissen CE (2021). Alzheimer's disease. Lancet.

[2] Villemagne VL, Burnham S, Bourgeat P, Brown B, Ellis KA, Salvado O (2013). Amyloid beta deposition, neurodegeneration, and cognitive decline in sporadic Alzheimer's disease: a prospective cohort study. Lancet Neurol.

[3] Imtiaz B, Tolppanen A-M, Kivipelto M, Soininen H (2014). Future directions in Alzheimer's disease from risk factors to prevention. Biochem Pharmacol.

[4] Cummings J, Lee G, Nahed P, Kambar M, Zhong K, Fonseca J (2022). Alzheimer's disease drug development pipeline: 2022. Alzheimers Dement (N Y).

[5] Gruters AAA, Ramakers I, Kessels RPC, Bouwman FH, OldeRikkert MGM, Blom MM (2019). Development of memory clinics in the Netherlands over the last 20 years. Int J Geriatr Psychiatry.

[6] Chen Y, Lebouvier T, Skrobala E, Volpe-Gillot L, Huvent-Grelle D, Jourdan N (2020). Twenty-year trends in patient referrals throughout the creation and development of a regional memory clinic network. Alzheimer's Dement: Translat Res Clin Interv.

[7] Kunneman M, Pel-Littel R, Bouwman FH, Gillissen F, Schoonenboom NSM, Claus JJ (2017). Patients' and caregivers' views on conversations and shared decision making in diagnostic testing for Alzheimer's disease: The ABIDE project. Alzheimers Dement (N Y).

[8] Visser LNC, Kunneman M, Murugesu L, van Maurik I, Zwan M, Bouwman FH (2019). Clinician-patient communication during the diagnostic workup: The ABIDE project. Alzheimers Dement (Amst).

[9] Fruijtier AD, Visser LNC, van Maurik IS, Zwan MD, Bouwman FH, van der Flier WM (2019). ABIDE Delphi study: topics to discuss in diagnostic consultations in memory clinics. Alzheimers Res Ther.

[10] Pinner G, Bouman WP (2003). What should we tell people about dementia?. Adv Psychiatr Treat.

[11] Grill JD, Cox CG, Kremen S, Mendez MF, Teng E, Shapira J (2017). Patient and caregiver reactions to clinical amyloid imaging. Alzheimers Dement.

[12] Dubois B, Feldman HH, Jacova C, Hampel H, Molinuevo JL, Blennow K (2014). Advancing research diagnostic criteria for Alzheimer's disease: the IWG-2 criteria. Lancet Neurol.

[13] Jack CR, Bennett DA, Blennow K, Carrillo MC, Dunn B, Haeberlein SB (2018). NIA-AA Research Framework: Toward a biological definition of Alzheimer's disease. Alzheimers Dement.

[14] Visser LNC, Minguillon C, Sanchez-Benavides G, Abramowicz M, Altomare D, Fauria K (2021). Dementia risk communication. A user manual for Brain Health Services-part 3 of 6. Alzheimers Res Ther..

[15] van der Flier WM, Kunneman M, Bouwman FH, Petersen RC, Smets EMA (2017). Diagnostic dilemmas in Alzheimer's disease: Room for shared decision making. Alzheimers Dement (N Y).

[16] van der Schaar J, Visser LNC, Bouwman FH, Ket JCF, Scheltens P, Bredenoord AL (2022). Considerations regarding a diagnosis of Alzheimer’s disease before dementia: a systematic review. Alzheimer’s Res Ther.

[17] Rothman AJ, Kiviniemi MT (1999). Treating people with information: an analysis and review of approaches to communicating health risk information. J Natl Cancer Inst Monogr.

[18] van Dulmen S (2011). The value of tailored communication for person-centred outcomes. J Eval Clin Pract.

[19] van Dyck CH, Swanson CJ, Aisen P, Bateman RJ, Chen C, Gee M (2022). Lecanemab in early Alzheimer’s disease. N Engl J Med.

[20] Ngandu T, Lehtisalo J, Solomon A, Levalahti E, Ahtiluoto S, Antikainen R (2015). A 2 year multidomain intervention of diet, exercise, cognitive training, and vascular risk monitoring versus control to prevent cognitive decline in at-risk elderly people (FINGER): a randomised controlled trial. Lancet.

[21] Survalyzer - to survey and analyze. Utrecht. 2018. p. https://survalyzer.com/.

[22] Degner LF, Sloan JA, Venkatesh P (1997). The Control Preferences Scale. Can J Nurs Res.

[23] Gerrity MS, White KP, DeVellis RF, Dittus RS (1995). Physicians' Reactions to Uncertainty: Refining the constructs and scales. Motiv Emot.

[24] Kunneman M, Smets EMA, Bouwman FH, Schoonenboom NSM, Zwan MD, Pel-Littel R (2017). Clinicians' views on conversations and shared decision making in diagnostic testing for Alzheimer's disease: The ABIDE project. Alzheimers Dement (N Y).

[25] Visser LNC, Pelt SAR, Kunneman M, Bouwman FH, Claus JJ, Kalisvaart KJ (2020). Communicating uncertainties when disclosing diagnostic test results for (Alzheimer’s) dementia in the memory clinic: The ABIDE project. Health Expect.

[26] Tarek ME, Segers K, Van Nechel C (2009). What Belgian neurologists and neuropsychiatrists tell their patients with alzheimer disease and why: a national survey. Alzheimer Dis Assoc Disord.

[27] Bertens D, Vos S, Kehoe P, Wolf H, Nobili F, Mendonca A (2019). Use of mild cognitive impairment and prodromal AD/MCI due to AD in clinical care: a European survey. Alzheimers Res Ther.

[28] Frederiksen KS, Nielsen TR, Appollonio I, Andersen BB, Riverol M, Boada M (2021). Biomarker counseling, disclosure of diagnosis and follow-up in patients with mild cognitive impairment: A European Alzheimer's disease consortium survey. Int J Geriatr Psychiatry.

[29] Nielsen TR, Svensson BH, Rohr G, Gottrup H, Vestergaard K, Hogh P (2020). The process of disclosing a diagnosis of dementia and mild cognitive impairment: a national survey of specialist physicians in Denmark. Dementia (London).

[30] Dubois B, Villain N, Frisoni GB, Rabinovici GD, Sabbagh M, Cappa S (2021). Clinical diagnosis of Alzheimer’s disease: recommendations of the International Working Group. Lancet Neurol.

[31] Knopman DS, Petersen RC, Jack CR (2019). A brief history of “Alzheimer disease”: Multiple meanings separated by a common name. Neurology.

[32] van Maurik IS, Vos SJ, Bos I, Bouwman FH, Teunissen CE, Scheltens P (2019). Biomarker-based prognosis for people with mild cognitive impairment (ABIDE): a modelling study. Lancet Neurol.

[33] Karikari TK, Pascoal TA, Ashton NJ, Janelidze S, Benedet AL, Rodriguez JL (2020). Blood phosphorylated tau 181 as a biomarker for Alzheimer's disease: a diagnostic performance and prediction modelling study using data from four prospective cohorts. Lancet Neurol.

[34] van der Flier WM, Scheltens P (2022). The ATN framework—moving preclinical Alzheimer disease to clinical relevance. JAMA Neurol.

[35] Ossenkoppele R, PichetBinette A, Groot C, Smith R, Strandberg O, Palmqvist S (2022). Amyloid and tau PET-positive cognitively unimpaired individuals are at high risk for future cognitive decline. Nat Med.

[36] Strikwerda-Brown C, Hobbs DA, Gonneaud J, St-Onge F, Binette AP, Ozlen H (2022). Association of elevated amyloid and tau positron emission tomography signal with near-term development of Alzheimer disease symptoms in older adults without cognitive impairment. JAMA Neurol.

[37] Ebenau JL, Timmers T, Wesselman LMP, Verberk IMW, Verfaillie SCJ, Slot RER (2020). ATN classification and clinical progression in subjective cognitive decline: The SCIENCe project. Neurology.

[38] van Maurik IS, Slot RER, Verfaillie SCJ, Zwan MD, Bouwman FH, Prins ND (2019). Personalized risk for clinical progression in cognitively normal subjects—the ABIDE project. Alzheimer’s Res Ther.

[39] Grill JD, Apostolova LG, Bullain S, Burns JM, Cox CG, Dick M (2017). Communicating mild cognitive impairment diagnoses with and without amyloid imaging. Alzheimer's Res Ther.

[40] Visser LNC, van Maurik IS, Bouwman FH, Staekenborg S, Vreeswijk R, Hempenius L (2020). Clinicians' communication with patients receiving a MCI diagnosis: The ABIDE project. PLoS ONE.

[41] Cantwell BM, Ramirez AJ (1997). Doctor-patient communication: a study of junior house officers. Med Educ.

[42] Moore PM, Rivera S, Bravo‐Soto GA, Olivares C, Lawrie TA. Communication skills training for healthcare professionals working with people who have cancer. Cochrane Database Syst Rev. 2018;(7):1–101. 10.1002/14651858.CD003751.pub4 PMC6513291 30039853

[43] Strout TD, Hillen M, Gutheil C, Anderson E, Hutchinson R, Ward H (2018). Tolerance of uncertainty: a systematic review of health and healthcare-related outcomes. Patient Educ Couns.

[44] Medendorp NM, Stiggelbout AM, Aalfs CM, Han PKJ, Smets EMA, Hillen MA (2021). A scoping review of practice recommendations for clinicians' communication of uncertainty. Health Expect.

[45] Rogers EM (1963). What are innovators like?. Theory into Pract.

[46] Tromp K, Smedinga M, Richard E, Perry M, Schermer MHN (2021). Views on early diagnosis of Alzheimer's disease among Dutch physicians: a qualitative interview study. J Alzheimers Dis.

[47] Rosenberg A, Ohlund-Wistbacka U, Hall A, Bonnard A, Hagman G, Ryden M (2022). beta-Amyloid, tau, neurodegeneration classification and eligibility for anti-amyloid treatment in a memory clinic population. Neurology.

[48] van Maurik IS, Visser LN, Pel-Littel RE, van Buchem MM, Zwan MD, Kunneman M (2019). Development and usability of ADappt: web-based tool to support clinicians, patients, and caregivers in the diagnosis of mild cognitive impairment and Alzheimer disease. JMIR Form Res.

[49] Bruun M, Frederiksen KS, Rhodius-Meester HFM, Baroni M, Gjerum L, Koikkalainen J (2019). Impact of a clinical decision support tool on dementia diagnostics in memory clinics: the PredictND validation study. Curr Alzheimer Res.

[50] van Gils AM, Visser LNC, Hendriksen HMA, Georges J, van der Flier WM, Rhodius-Meester HFM (2022). Development and design of a diagnostic report to support communication in dementia: Co-creation with patients and care partners. Alzheimers Dement (Amst).

[51] Largent EA, Grill J, O'Brien K, Wolk D, Harkins K, Karlawish J (2023). Testing for Alzheimer disease biomarkers and disclosing results across the disease continuum. Neurology.

[52] Van Gils AM, Visser LNC, Hendriksen HMA, Georges J, Muller M, Bouwman FH (2021). The (non)sense of diagnostic computer tools in memory clinics: An international survey assessing the views of clinicians, patients and caregivers. Alzheimers Dement.

[53] Bos-van den Hoek DW, Visser LNC, Brown RF, Smets EMA, Henselmans I. Communication skills training for healthcare professionals in oncology over the past decade: a systematic review of reviews. Curr Opin Support Palliat Care. 2019;13(1):33–45. 10.1097/SPC.0000000000000409 30562180

