# Supplementary material for: Communication about diagnosis, prognosis, and prevention in the memory clinic: perspectives of European memory clinic professionals
Source: Alzheimers Res Ther. 2023 Aug 5;15:131. doi: 10.1186/s13195-023-01276-9 (PMC10404377; doi:10.1186/s13195-023-01276-9)
Supplement: Supplementary file 1 — Additional file 1. Survey. The perspectives of memory clinic clinicians on (communicating about) early diagnosis of Alzheimer’s disease, dementia risk and prevention: a EU-FINGERS & LETHE survey. [file 13195_2023_1276_MOESM1_ESM.pdf]

Welcome!

The perspectives of memory clinic clinicians on (communicating about) early diagnosis of Alzheimer's disease, dementia risk and prevention: a EU-FINGERS & LETHE survey.

Dear colleague,

We kindly ask you to participate in an online survey study titled "**The perspectives of memory clinic clinicians on (communicating about) early diagnosis of Alzheimer's disease, dementia risk and prevention: a EU-FINGERS & LETHE survey**" among clinicians working in European memory clinics. This research is carried out by Alzheimer center Amsterdam, The Netherlands, in collaboration with Karolinska Insitututet, Sweden, as part of the EU-FINGERS (EU Joint Programme - Neurodegenerative Disease Research, JPND, www.eufingers.com) and LETHE (European Union's Horizon 2020, www.lethe-project.eu) projects. The ethics committee of the Amsterdam UMC has reviewed the study protocol and materials, and approved that this study is not subject to the Medical Research Involving Human Subjects Act (WMO).

Questions?
If you have questions about this study, please do not hesitate to contact us at any time.

Thank you very much in advance, also on behalf of Leonie Visser,

PhD, post-doctoral researcher at Center for Alzheimer Research, Karolinska Institutet, and the EU-FINGERS and LETHE consortia,

Heleen M.A. Hendriksen, MSc.
PhD student and neuropsychologist, Alzheimer center Amsterdam
h.hendriksen@amsterdamumc.nl
+31 20 4440685

Please click '**Next**' to learn about the background and aim of the study

Background and aim of the research

Background and aim of the study

Alzheimer's disease (AD) is the most common cause of dementia, with no existing cure yet. AD develops over a period of 20-30 years, gradually progressing to dementia. Prevention of dementia is crucial and research therefore focusses on the early stages of AD. Biomarkers have been developed to detect AD-related pathological processes, measured through neuro-imaging (MRI), cerebrospinal fluid (CSF), or amyloid PET.

In patients with dementia, such diagnostic tests are used to assess whether AD is the underlying pathology[1]. For individuals with subjective cognitive decline (SCD) or mild cognitive impairment (MCI), AD biomarker testing may have complex implications. Nevertheless, increasing numbers of patients with SCD and MCI (and their care partners) visit memory clinics with a high need for information about diagnostic testing, test results and the consequences thereof for their daily life[2,3]. To attune memory clinic care to their individual needs, clinician-patient communication is key, yet complex. It is important to develop best practice strategies for communicating about an (early) AD diagnosis, dementia risk and prevention with patients and their care partners.

Our aim is to survey clinicians' perspectives on early AD biomarker testing, dementia risk and preventive efforts, including their thoughts on how to communicate about these topics with patients and their care partners. We are specifically aiming to collect the views of clinicians, e.g., physicians, nurses, psychologists, involved in the diagnostic work-up for dementia at a hospital/memory clinic. Since differences in attitudes, experiences, and opinions are likely to exist amongst countries[4], for example due to differences in health care systems and cultural background, we collect data internationally to compare across European countries.

Please click '**Next**' to provide digital informed consent

Literature

1.Jack CR, Jr., Albert MS, et al. Introduction to the recommendations from the National Institute on Aging-

Alzheimer's Association workgroups on diagnostic guidelines for Alzheimer's disease. *Alzheimer's & dementia : the journal of the Alzheimer's Association*. 2011;7(3):257.
2.Kunneman M, Pel-Littel R, et al. Patients' and caregivers' views on conversations and shared decision making in diagnostic testing for Alzheimer's disease: The ABIDE project. *Alzheimers Dement (N Y)*. 2017;3(3):314.
3.Visser LNC, Kunneman M, et al. Clinician-patient communication during the diagnostic workup: The ABIDE project. *Alzheimers Dement (Amst)*. 2019;11:520.
4.Kunneman M, Smets EMA, et al. Clinicians' views on conversations and shared decision making in diagnostic testing for Alzheimer's disease: The ABIDE project. *Alzheimers Dement (N Y)*. 2017;3(3):305.

Digital informed consent

Digital informed consent for participation in the online survey

Informedconsent

Use and storage of your data

Your responses to this survey are anonymous, i.e., we will not ask you to provide any personal data such as your name, email address or date of birth. Data will be kept confidential. After closure of the study, data are stored for 15 years. Data will be stored at a protected drive of the Neurology department of Amsterdam UMC in the Netherlands.

- ☐ By ticking this box, you provide digital informed consent for using the anonymous data you will provide

Practical information

Thank you for participating in our EU-FINGERS & LETHE survey!
The survey consists of 4 parts:

Part 1 – Background information
Part 2 – Organization of the memory clinic and diagnostic procedures
Part 3 – Clinician-patient communication – cases
Part 4 – Clinician-patient communication – additional topics

Since we collect the data through an anonymous link, it is important to complete the survey in one sitting. This will take approximately 20 minutes. You can see your progress on the top of the screen.

Please click '**Next**' to continue

Before you start, we would like to share the following definitions we used:

- Clinician: a health care professional, e.g., a physician, nurse, psychologist, etc.

- Memory clinic: a clinic/hospital department specialized in the diagnostic work-up for dementia, i.e., providing diagnostic care for individuals with cognitive symptoms and/or concerns about their cognitive functioning.

- Biomarkers in the context of Alzheimer's disease: β-amyloid (Aβ) deposition, pathologic tau, and neurodegeneration.

- Care partner: refers to a close relative or friend of the patient, who provides emotional and/or practical support, e.g., by accompanying the patient to healthcare appointments.

- Shared decision making: a communication process in which both the patient and clinician contribute to the medical decision-making process and agree on diagnostic/treatment decisions.

Part 1 - Background information

Part 1 – Background information

What is your gender?

Gender

- Female
- Male
- I prefer not to answer

What is your age?

Age

- 18-30 years old
- 31-40 years old
- 41-50 years old
- 51-60 years old
- 61-70 years old
- 71-80 years old

This survey is aimed at clinicians (e.g., physicians, nurses, psychologists) **who have clinical experience in diagnosing dementia, i.e., who are involved in the diagnostic work up for dementia. Does this apply to you?**

Exclusioncriteria

- Yes, I am currently involved as a clinician in the diagnostic work up for dementia
- Yes, I used to be involved as a clinician in the diagnostic work up for dementia
- No, I do not have clinical experience with regard to the diagnostic work up for dementia

Dit element tonen

Als This survey is aimed at clinicians (e.g., physicians, nurses, psyc... with regard to the diagnostic work up for dementia

No, I do not have clinical experience
Is geselecteerd

Thank you for your time and interest.

Please click 'Next' to end the survey.

Dit element tonen

Als This survey is aimed at clinicians (e.g., physicians, nurses, psyc... with regard to the diagnostic work up for dementia

No, I do not have clinical experience
Is niet geselecteerd

How many years of experience do you have in the diagnostic work up for dementia?

Yearsofexperience

____

Dit element tonen

Als This survey is aimed at clinicians (e.g., physicians, nurses, psyc... with regard to the diagnostic work up for dementia — No, I do not have clinical experience Is niet geselecteerd

In which country do you currently work?
Multiple answers can be selected

Country

- ▫ The Netherlands
- ▫ Sweden
- ▫ Finland
- ▫ Germany
- ▫ Spain
- ▫ Hungary
- ▫ Luxemburg
- ▫ Italy
- ▫ Austria
- ▫ Other, namely:_____

Dit element tonen

Als This survey is aimed at clinicians (e.g., physicians, nurses, psyc... with regard to the diagnostic work up for dementia — No, I do not have clinical experience Is niet geselecteerd

What is your profession?

Profession

- ○ Physician, after completion of specialist training
- ○ Physician, currently in specialist training
- ○ Physician, not in/without specialist training
- ○ (Specialized) Nurse or nurse practitioner
- ○ Neuropsychologist
- ○ Other, namely:_____

Dit element tonen

Als This survey is aimed at clinicians (e.g., physicians, nurses, psyc... with regard to the diagnostic work up for dementia — No, I do not have clinical experience Is niet geselecteerd

What medical specialty do you mainly work in?

Specialty

- ○ Neurology
- ○ Clinical geriatric medicine
- ○ Psychiatry
- ○ Internal (geriatric) medicine
- ○ Other, namely:_____

Dit element tonen

Als This survey is aimed at clinicians (e.g., physicians, nurses, psyc... with regard to the diagnostic work up for dementia

No, I do not have clinical experience
Is niet geselecteerd

In what type of organization/hospital are you currently working?

Organization

- Academic/university hospital
- Non-academic teaching hospital
- Non-teaching hospital
- Mental health service
- Other, namely:_____

Dit element tonen

Als This survey is aimed at clinicians (e.g., physicians, nurses, psyc... clinician in the diagnostic work up for dementia

Yes, I am currently involved as a
Is geselecteerd

How many memory clinic patients do <u>you</u> see on average per month?

nrpatientsclinician

____

Dit element tonen

Als This survey is aimed at clinicians (e.g., physicians, nurses, psyc... clinician in the diagnostic work up for dementia

Yes, I am currently involved as a
Is geselecteerd

How many <u>new</u> memory clinic patients do <u>you</u> see on average per month?

nrnewpatientsclinician

____

Dit element tonen

Als This survey is aimed at clinicians (e.g., physicians, nurses, psyc... with regard to the diagnostic work up for dementia

No, I do not have clinical experience
Is niet geselecteerd

Part 2 - Organization of the memory clinic and diagnostic procedures

Part 2 – Organization of the memory clinic and diagnostic procedures

How many new patients visit your memory clinic each month?
(If not sure, please provide an estimate) nrnewpatientsclinic

____

Which disciplines are involved in your memory clinic?

Multiple answers can be selected Disciplines

- □ Neurology
- □ Clinical geriatric medicine
- □ Psychiatry
- □ Internal (geriatric) medicine
- □ (Neuro)psychology
- □ Nursing
- □ Radiology
- □ Clinical Neurophysiology
- □ Nuclear medicine
- □ Speech therapy
- □ Other, namely:_____

Does your clinic have a multidisciplinary diagnostic meeting, i.e., a meeting in which clinicians from different medical specialties discuss and diagnose patients together?

MDM

- ○ Yes
- ○ No

How long is the average period from first visit to the consultation in which test results/diagnosis are disclosed?

Perioddiagnosis

- ○ A few days
- ○ 1-2 weeks
- ○ 3-4 weeks
- ○ 5-6 weeks
- ○ 7-8 weeks
- ○ 9-10 weeks
- ○ 11-12 weeks
- ○ 3-4 months
- ○ 5-6 months
- ○ More than 6 months

In case of a (neurodegenerative) diagnosis, is there a routine follow up scheduled after the disclosure consultation, with a physician or another clinician at the clinic? routinefollowup

- Yes, within a month
- Yes, between 1-7 months
- Yes, between 8-13 months
- Yes, after more than 13 months
- No, only on request of the patient
- No

Which tests can be performed at your memory clinic?

Multiple answers can be selected Diagnostictests

- ◻ Lab/blood work
- ◻ Neuropsychological testing
- ◻ CT scan
- ◻ MRI scan
- ◻ Cerebrospinal fluid (CSF) biomarkers
- ◻ Amyloid PET scan
- ◻ FDG PET scan
- ◻ EEG
- ◻ Genetic testing
- ◻ Consultation with speech therapist
- ◻ Consultation with psychiatrist
- ◻ Consultation with occupational therapist
- ◻ Other, namely:_____

Dit element tonen

Als Which tests can be performed at your memory clinic?
Multiple answe... Genetic testing Is geselecteerd

Please specify genetic testing

Multiple answers can be selected Genetictesting

- ◻ APOE
- ◻ A core gene panel for familiar AD and/or FTD (e.g. PSEN1, GRN, MAPT)
- ◻ A dementia gene panel based on exome sequencing
- ◻ C9ORF72 repeat length analysis
- ◻ I don't know

How is the diagnostic work-up organized? Protocol

- ○ A standardized protocol, i.e., in essence, every patient undergoes the same battery of tests.
- ○ A semi-standardized protocol, i.e., 1) we have a standardized basic set of tests (e.g. lab work and neuropsychological assesment) and per patient additional tests can be requested (e.g. MRI or lumbar puncture) **or** 2) we have multiple patient journeys for different patient categories.
- ○ A personalized protocol, i.e., diagnostics tests are selected for each individual patient.

Dit element tonen

Als How is the diagnostic work-up organized? A personalized protocol, i.e., diagnostics tests are selected for each individual patient. Is niet geselecteerd

If standardized/semi-standardized, what does the routine protocol consist of?

Multiple answers can be selected Routineprotocol

- ◻ (hetero)Anamnesis
- ◻ Neurological examination
- ◻ Lab/blood work
- ◻ Neuropsychological testing
- ◻ CT scan
- ◻ MRI scan
- ◻ Cerebrospinal fluid (CSF) biomarkers
- ◻ Amyloid PET scan
- ◻ FDG PET scan
- ◻ EEG
- ◻ Genetic testing
- ◻ Consultation with speech therapist
- ◻ Consultation with psychiatrist
- ◻ Consultation with occupational therapist
- ◻ Other, namely:_____

Dit element tonen

Als If standardized/semi-standardized, what does the routine protocol ... Genetic testing Is geselecteerd

Please specify genetic testing

Multiple answers can be selected Geneticstandardized

- ◻ APOE
- ◻ A core gene panel for familiar AD and/or FTD (e.g. PSEN1, GRN, MAPT)
- ◻ A dementia gene panel based on exome sequencing
- ◻ C9ORF72 repeat length analysis
- ◻ I don't know

Dit element tonen

Als How is the diagnostic work-up organized? A standardized protocol, i.e., in essence, every patient undergoes the same battery of tests. Is niet geselecteerd

If not standardized, on what factors is the decision on what diagnostic tests to perform based?

Multiple answers can be selected Decisiondiagnostictests

- ▫ The patient’s preferences
- ▫ The care partner’s preferences
- ▫ Age of the patient
- ▫ Gender of the patient
- ▫ Results on cognitive screening tests (MMSE / MoCA / Addenbrooke's Cognitive Examination / CERAD)
- ▫ Symptom presentation / anamnestic information
- ▫ Referral information
- ▫ Comorbidity factors
- ▫ National clinical guidelines
- ▫ International clinical guidelines
- ▫ Resources in terms of insurance/payment
- ▫ Resources in terms of availability of diagnostic tests or personnel
- ▫ Participation in research study
- ▫ Other, namely:_____

To what extent is the decision about whether or not to perform a MRI/CT to detect neurodegeneration already made before the patient’s first visit to the memory clinic, i.e., upon referral?

(0 = not at all, 10 = completely) Decisionmrict

- ○ 0
  Not at all
- ○ 1
- ○ 2
- ○ 3
- ○ 4
- ○ 5
- ○ 6
- ○ 7
- ○ 8
- ○ 9
- ○ 10
  Completely

To what extent is the decision about whether or not to perform a lumbar puncture or PET-scan to detect β-amyloid (Aβ) deposition and/or pathologic tau already made before the patient's first visit to the memory clinic, i.e., upon referral?
(0 = not at all, 10 = completely)

Decisionamyloidtau

- 0
  Not at all
- 1
- 2
- 3
- 4
- 5
- 6
- 7
- 8
- 9
- 10
  Completely

Who has the biggest say in decisions about biomarker testing for Alzheimer's disease in your memory clinic? biggestsay

- These decisions are made at memory clinic level, since our clinic adopts a standardized protocol for testing
- The medical doctor who's responsible/the treating physician
- The multidisciplinary team
- The patient and his/her care partner
- The referring medical doctor
- Other, namely:_____

Which individual diagnostic test result(s) do you disclose to your patient (if test is performed)?

Multiple answers can be selected Disclosurediagnostictests

- ◻ This differs per patient
- ◻ I do not disclose test results
- ◻ Lab/blood work
- ◻ Neuropsychological testing
- ◻ CT scan
- ◻ MRI scan
- ◻ Cerebrospinal fluid (CSF) biomarkers
- ◻ Amyloid PET scan
- ◻ FDG PET scan
- ◻ EEG
- ◻ Genetic testing

Dit element tonen

Als	Which individual diagnostic test result(s) do you disclose to your...	Genetic testing	Is geselecteerd

Please specify genetic testing

Multiple answers can be selected Genetictestingdisclosure

- ◻ APOE
- ◻ A core gene panel for familiar AD and/or FTD (e.g. PSEN1, GRN, MAPT)
- ◻ A dementia gene panel based on exome sequencing
- ◻ C9ORF72 repeat length analysis

Dit element tonen

Als Which individual diagnostic test result(s) do you disclose to your... This differs per patient Is geselecteerd

If it differs per patient which diagnostic test result(s) you disclose, on what factors is that decision based?

Multiple answers can be selected Differsperpatient

- ◻ Diagnosis of the patient
- ◻ The patient's preferences
- ◻ The care partner's preferences
- ◻ Age of the patient
- ◻ Gender of the patient
- ◻ Results on cognitive screening tests (MMSE / MoCA / Addenbrooke's Cognitive Examination / CERAD)
- ◻ Symptom presentation / anamnestic information
- ◻ Referral information
- ◻ Comorbidity factors
- ◻ National clinical guidelines
- ◻ International clinical guidelines
- ◻ Resources in terms of insurance/payment
- ◻ Resources in terms of availability of diagnostic tests or personnel
- ◻ Other, namely:_____

Dit element tonen

Als If it differs per patient which diagnostic test result(s) you disc... The patient's preferences Is geselecteerd

If it is (partly) based on the patient's preferences which diagnostic test result(s) you disclose, when are these preferences discussed? Patientpreferenceswhen

- o Before the first consultation
- o During the first consultation
- o During the consultation in which the diagnostic test result(s) are disclosed
- o Other, namely:_____

Dit element tonen

Als If it differs per patient which diagnostic test result(s) you disc... The patient's preferences Is geselecteerd

By whom are these preferences discussed? patientpreferenceswhom

- o The memory clinic treating medical doctor
- o The memory clinic nurse
- o The general practitioner/primary care physician
- o The referring medical doctor
- o Other, namely:_____

Do you use tools to support the conversation with your patients, for example to help you explain the procedure of the consultation or to help you explain the test results (e.g. visual display/brain images, risk calculation tool, drawings, etc.)? Tools

- o Yes, with most/all patients
- o Yes, with some patients
- o Yes, but only if patients or their care partners would ask for it
- o No

Dit element tonen

Als Do you use tools to support the conversation with your patients, f... No Is niet geselecteerd

If yes, which tools do you use? whichtools

____

Do you provide your patients with written materials (to take home)? writtenmaterials

- No
- Yes, with generic/general information
- Yes, a personalized text (e.g. a summary)
- Yes, both generic/general information and a personalized text (e.g. a summary)

Dit element tonen

Als This survey is aimed at clinicians (e.g., physicians, nurses, psyc... with regard to the diagnostic work up for dementia | No, I do not have clinical experience Is niet geselecteerd

Part 3. Clinician-patient communication - cases

Part 3 - Clinician-patient communication - cases
On the following pages we provide you with 5 patient cases. Per case, we would like to know your opinion on how to communicate diagnosis and prognosis during the disclosure consultation to the hypothetical patient described in the case. So, these cases are **not** meant to test if you would come to the 'right' conclusion regarding the diagnosis. Instead, we would like to know what you would tell these patients and their care partners, **even if you normally do not disclose test results.**

Please click '**Next**' for the first patient case

A 72 year old woman was referred to a memory clinic because of worsening memory impairment and difficulty finding words. Her husband helps her to get dressed in the morning. He feels uncomfortable leaving her alone at home. The result on the Mini-Mental State Examination (MMSE) is 16/30. The neuropsychological examination shows impairments in memory, language and visuospatial abilities. The MRI shows bilateral medial temporal lobe atrophy (MTA 3) and bilateral parietal atrophy (PA 2). Cerebrospinal fluid measures amyloid-beta 42, total tau and phosphorylated tau are abnormal.

Would you communicate *dementia* as the patient's syndrome diagnosis?

ptcase1diagnosis

- Yes
- Yes, but I would only communicate a syndrome diagnosis if the patient prefers to know
- No, I would not communicate any syndrome diagnosis or any diagnostic label (I would summarize test results in my own words)
- No, I would communicate another syndrome diagnosis/label, namely….______

Would you communicate Alzheimer's disease as the underlying pathology to this patient?

ptcase1alzheimer

- Yes
- No

Would you explain the difference between dementia and Alzheimer's disease?

ptcase1difference

- Yes
- No

Would you communicate about prognosis in terms of symptom progression to this patient, i.e. explain that symptoms will get worse over time? ptcase1prognosis

- ○ Yes
- ○ Only if the patient or her partner prefers to know
- ○ No

Would you communicate about the following aspects of post diagnostic-support with this patient during the disclosure consultation, such as: ptcase1support

	Yes	No
Counselling by a specialized nurse?	○	○
Peer support groups?	○	○
Disease specific organizations, e.g. Alzheimer’s associations?	○	○
Clinical trial opportunities?	○	○
Information on driving?	○	○
Advanced care planning?	○	○
Case management?	○	○
Informational websites?	○	○

Would you communicate about prevention in attempt to delay further cognitive decline with this patient? ptcase1lifestyle

- ○ Yes
- ○ Only if the patient or her partner prefers to know
- ○ No

Dit element tonen

Als Would you communicate about prevention in attempt to delay further... No Is niet geselecteerd

If yes, what would you discuss as potential means to reduce cognitive decline?
I address:
Multiple answers can be selected ptcase1potentialmeans

- ◻ Diet and nutrition
- ◻ Exercise
- ◻ Keeping the brain active (cognitively stimulating activities)
- ◻ Medication for modifiable risk factors such as hypertension or diabetes
- ◻ Quit smoking
- ◻ More moderate alcohol consumption
- ◻ Reduce stress
- ◻ Sleep
- ◻ Maintaining social contacts and relationships
- ◻ All of the above
- ◻ Other, namely:_____

Dit element tonen

Als Would you communicate about prevention in attempt to delay further... No Is niet geselecteerd

Would you personalize this preventive strategy? ptcase1personalize

- ○ No
- ○ Yes

Dit element tonen

Als Would you personalize this preventive strategy? Yes Is geselecteerd

If yes, based on what?
Multiple answers can be selected ptcase1personalizeyes

- ◻ Demographics
- ◻ Diagnostic test results (e.g. cognitive tests, biomarkers)
- ◻ Anamnestic information (e.g., what they have told about lifestyle)

Dit element tonen

Als	Would you communicate about prevention in attempt to delay further...	No	Is geselecteerd

Why not?
Multiple answers can be selected ptcase1lifestyleno

- ▫ I don't know enough about this topic
- ▫ This is not relevant for this patient
- ▫ I don't think my patients are interested in this type of information
- ▫ It is difficult to talk about this without accusing/putting the blame on the patient
- ▫ This topic is of lower priority and usually there is no time to discuss this during the consultation
- ▫ Too speculative/there is not yet enough evidence about effective prevention strategies
- ▫ There are no guidelines which I can follow
- ▫ Other, namely:_____

A 63 year old man was referred to a memory clinic because he experiences a lot of stress during his work as a teacher. He finds it hard to remember all his student's names and to learn how to work with a new digital module. Family has not noticed, but when asked, the colleagues remember that he forgot some meetings, which was not like him at all. MMSE is 26/30. The neuropsychological examination shows memory impairments, the test scores on all other cognitive domains are within limits. The MRI shows no abnormalities. Cerebrospinal fluid measures amyloid-beta 42, total tau and phosphorylated tau are normal.

Would you communicate Mild Cognitive Impairment (MCI) as the patient's syndrome diagnosis/diagnostic label? ptcase2diagnosis

- o Yes
- o Yes, but I would only communicate a syndrome diagnosis if the patient prefers to know
- o No, I would not communicate any syndrome diagnosis or any diagnostic label (I would summarize test results in my own words)
- o No, I would communicate another syndrome diagnosis/diagnostic label, namely:_____

Would you communicate the biomarker results to this patient? ptcase2biomarker

- Yes, I would communicate the biomarker results, yet emphasize that we do not know exactly what this means for the patient
- Yes, I would communicate the biomarker results and tell that the patient currently does not have Alzheimer's disease
- No, I would not communicate the biomarker results

Dit element tonen

Als Would you communicate the biomarker results to this patient? No, I would not communicate the biomarker results Is geselecteerd

If not, why? I would not disclose because…
Multiple answers can be selected ptcase2biomarkerno

- ◻ It is not included in national care guidelines
- ◻ Disclosure may upset/worry the patient (and/or care partner)
- ◻ The information is too difficult for the patient to understand
- ◻ I am not sure how to interpret the results myself
- ◻ There is no added value over a clinical diagnosis
- ◻ There are no implications for the prognosis of an individual patient
- ◻ I feel not equipped to disclose these results
- ◻ Other, namely:_____

Would you communicate prognosis in terms of dementia risk to this patient? ptcase2prognosis

- ○ Yes
- ○ Only if the patient or his partner prefers to know
- ○ No

Dit element tonen

Als Would you communicate prognosis in terms of dementia risk to this ... No Is niet geselecteerd

If so, would you personalize this risk? ptcase2personalizerisk

- ○ No, I would just explain that individuals with MCI have a higher chance of developing dementia
- ○ No, I would explain the chance of developing dementia is fifty-fifty, in this case
- ○ Yes

Dit element tonen

Als If so, would you personalize this risk? Yes Is geselecteerd

If yes, based on what?
Multiple answers can be selected ptcase2personalizeriskyes

- ◻ Demographics
- ◻ Diagnostic test results (i.e., in this case, negative biomarkers indicate a low risk of developing dementia due to Alzheimer's disease)
- ◻ Anamnestic information
- ◻ My experience

Would you communicate about the following aspects of post diagnostic-support with this patient <u>during the disclosure consultation</u>, such as:

ptcase2support

	Yes	No
Counselling by a specialized nurse?	○	○
Peer support groups?	○	○
Disease specific organizations, e.g. Alzheimer’s associations?	○	○
Clinical trial opportunities?	○	○
Information on driving?	○	○
Advanced care planning?	○	○
Case management?	○	○
Informational websites?	○	○

Would you communicate about prevention of dementia or cognitive decline with this patient?

ptcase2prevention

- ○ Yes
- ○ Only if the patient or his partner prefers to know
- ○ No

Dit element tonen

Als Would you communicate about prevention of dementia or cognitive de... No Is niet geselecteerd

If yes, what would you discuss as potential means to reduce the risk of dementia/cognitive decline? I address:

Multiple answers can be selected ptcase2means

- □ Diet and nutrition
- □ Exercise
- □ Keeping the brain active (cognitively stimulating activities)
- □ Medication for modifiable risk factors such as hypertension or diabetes
- □ Quit smoking
- □ More moderate alcohol consumption
- □ Reduce stress
- □ Sleep
- □ Maintaining social contacts and relationships
- □ All of the above
- □ Other, namely:_____

Dit element tonen

Als Would you communicate about prevention of dementia or cognitive de... No Is niet geselecteerd

Would you personalize this preventive strategy? ptcase2personalize

- ○ No
- ○ Yes

Dit element tonen

Als Would you personalize this preventive strategy? Yes Is geselecteerd

If yes, based on what?

Multiple answers can be selected ptcase2personalizeyes

- □ Demographics
- □ Diagnostic test results (e.g. cognitive tests, biomarkers)
- □ Anamnestic information (e.g., what they have told about lifestyle)

Dit element tonen

Als	Would you communicate about prevention of dementia or cognitive de...	No	Is geselecteerd

Why not?
Multiple answers can be selected ptcase2lifestyleno

- ▫ I don't know enough about this topic
- ▫ This is not relevant for this patient
- ▫ I don't think my patients are interested in this type of information
- ▫ It is difficult to talk about this without accusing/putting the blame on the patient
- ▫ This topic is of lower priority and usually there is no time to discuss this during the consultation
- ▫ Too speculative/there is not yet enough evidence about effective prevention strategies
- ▫ There are no guidelines which I can follow
- ▫ Other, namely:_____

____

Consider the previous patient, but now the MRI shows medial temporal lobe atrophy (MTA) of 2 on the left and 1 on the right. Cerebrospinal fluid measures amyloid-beta 42, total tau and phosphorylated tau are abnormal.

(A 63 year old man was referred to a memory clinic because he experiences a lot of stress during his work as a teacher. He finds it hard to remember all his student's names and to learn how to work with a new digital module. Family has not noticed, but when asked, the colleagues remember that he forgot some meetings, which was not like him at all. MMSE is 26/30. The neuropsychological examination shows memory impairments, the test scores on all other cognitive domains are within limits.)

Would you communicate Mild Cognitive Impairment (MCI) as the patient's syndrome diagnosis/diagnostic label? ptcase3diagnosis

- o Yes
- o Yes, but I would only communicate a syndrome diagnosis if the patient prefers to know
- o No, I would not communicate any syndrome diagnosis or any diagnostic label (I would summarize test results in my own words)
- o No, I would communicate another syndrome diagnosis/diagnostic label, namely:_____

Would you communicate the biomarker results to this patient? ptcase3biomarker

- Yes, I would communicate the biomarker results, yet emphasize that we do not know exactly what this means for the patient
- Yes, I would communicate that the biomarker results imply the presence of Alzheimer’s disease
- No, I would <u>not</u> communicate the biomarker results

Dit element tonen

Als Would you communicate the biomarker results to this patient? No, I would not communicate the biomarker results Is geselecteerd

If not, why? I would not disclose because…
Multiple answers can be selected ptcase3biomarkerno

- ◻ It is not included in national care guidelines
- ◻ Disclosure may upset/worry the patient (and/or care partner)
- ◻ The information is too difficult for the patient to understand
- ◻ I am not sure how to interpret the results myself
- ◻ There is no added value over a clinical diagnosis
- ◻ There are no implications for the prognosis of an individual patient
- ◻ I feel not equipped to disclose these results
- ◻ Other, namely:_____

Would you communicate prognosis in terms of dementia risk to this patient? ptcase3prognosis

- ○ Yes
- ○ Only if the patient or his partner prefers to know
- ○ No

Dit element tonen

Als Would you communicate prognosis in terms of dementia risk to this ... No Is niet geselecteerd

If so, would you personalize this risk? ptcase3personalizerisk

- ○ No, I would just explain that individuals with MCI have a higher chance of developing dementia
- ○ No, I would explain the chance of developing dementia is fifty-fifty, in this case
- ○ Yes

Dit element tonen

Als If so, would you personalize this risk? Yes Is geselecteerd

If yes, based on what?
Multiple answers can be selected ptcase3personalizeriskyes

- ◻ Demographics
- ◻ Diagnostic test results (i.e., in this case, positive biomarkers indicate a high risk of developing dementia due to Alzheimer's disease)
- ◻ Anamnestic information
- ◻ My experience

Would you communicate about the following aspects of post diagnostic-support with this patient <u>during the disclosure consultation</u>, such as:

ptcase3support

	Yes	No
Counselling by a specialized nurse?	○	○
Peer support groups?	○	○
Disease specific organizations, e.g. Alzheimer's associations?	○	○
Clinical trial opportunities?	○	○
Information on driving?	○	○
Advanced care planning?	○	○
Case management?	○	○
Informational websites?	○	○

Would you communicate about prevention of dementia or cognitive decline with this patient?

ptcase3prevention

- ○ Yes
- ○ Only if the patient or his partner prefers to know
- ○ No

Dit element tonen

Als Would you communicate about prevention of dementia or cognitive de... No Is niet geselecteerd

If yes, what would you discuss as potential means to reduce the risk of dementia/cognitive decline? I address:
Multiple answers can be selected ptcase3means

- ◻ Diet and nutrition
- ◻ Exercise
- ◻ Keeping the brain active (cognitively stimulating activities)
- ◻ Medication for modifiable risk factors such as hypertension or diabetes
- ◻ Quit smoking
- ◻ More moderate alcohol consumption
- ◻ Reduce stress
- ◻ Sleep
- ◻ Maintaining social contacts and relationships
- ◻ All of the above
- ◻ Other, namely:_____

Dit element tonen

Als Would you communicate about prevention of dementia or cognitive de... No Is niet geselecteerd

Would you personalize this preventive strategy? ptcase3personalize

- ○ No
- ○ Yes

Dit element tonen

Als Would you personalize this preventive strategy? Yes Is geselecteerd

If yes, based on what?
Multiple answers can be selected ptcase3personalizeyes

- ◻ Demographics
- ◻ Diagnostic test results (e.g. cognitive tests, biomarkers)
- ◻ Anamnestic information (e.g., what they have told about lifestyle)

Dit element tonen

Als Would you communicate about prevention of dementia or cognitive de... No Is geselecteerd

Why not?

Multiple answers can be selected ptcase3preventionno

- ▫ I don't know enough about this topic
- ▫ This is not relevant for this patient
- ▫ I don't think my patients are interested in this type of information
- ▫ It is difficult to talk about this without accusing/putting the blame on the patient
- ▫ This topic is of lower priority and usually there is no time to discuss this during the consultation
- ▫ Too speculative/there is not yet enough evidence about effective prevention strategies
- ▫ There are no guidelines which I can follow
- ▫ Other, namely:_____

A 59 year old woman was referred to the memory clinic at her own request by her general practitioner. She experiences memory problems, such as forgetting her keys. She feels a lot of anxiety at these moments. She works part-time as a social worker. MMSE is 29/30. The neuropsychological test scores are all within limits. The MRI shows no abnormalities. Cerebrospinal fluid measures amyloid-beta 42, total tau and phosphorylated tau are <u>normal</u>.

Would you communicate Subjective Cognitive Decline (SCD) as the patient's syndrome diagnosis/diagnostic label? ptcase4diagnosis

- o Yes
- o Yes, but I would only communicate a syndrome diagnosis if the patient prefers to know
- o No, I would <u>not</u> communicate any syndrome diagnosis or any diagnostic label (I would summarize test results in my own words)
- o No, I would communicate another syndrome diagnosis/diagnostic label, namely:_____

Would you communicate the biomarker results to this patient? ptcase4biomarker

- o Yes, I would communicate the biomarker results, yet emphasize that we do not know exactly what this means for the patient
- o Yes, I would communicate the biomarker results and tell that the patient currently does not have Alzheimer's disease

- No, I would not communicate the biomarker results

Dit element tonen

Als Would you communicate the biomarker results to this patient? No, I would not communicate the biomarker results Is geselecteerd

If not, why? I would not disclose because…
Multiple answers can be selected ptcase4biomarkerno

- ◻ It is not included in national care guidelines
- ◻ Disclosure may upset/worry the patient (and/or (care) partner)
- ◻ The information is too difficult for the patient to understand
- ◻ I am not sure how to interpret the results myself
- ◻ There is no added value over a clinical diagnosis
- ◻ There are no implications for the prognosis of an individual patient
- ◻ I feel not equipped to disclose these results
- ◻ Other, namely:_____

Would you communicate prognosis in terms of dementia risk to this patient?
ptcase4prognosis

- ○ Yes
- ○ Only if the patient or his partner prefers to know
- ○ No

Dit element tonen

Als Would you communicate prognosis in terms of dementia risk to this ... No Is niet geselecteerd

If so, would you personalize this risk?
ptcase4personalizerisk

- ○ No
- ○ Yes

Dit element tonen

Als If so, would you personalize this risk? Yes Is geselecteerd

If yes, based on what?
Multiple answers can be selected pcase4personalizeriskyes

- ◻ Demographics
- ◻ Diagnostic test results (i.e., in this case, negative biomarkers indicate a low risk of developing dementia due to Alzheimer's disease)
- ◻ Anamnestic information
- ◻ My experience

Would you communicate about prevention of dementia or cognitive decline with this patient? ptcase4prevention

- ○ Yes
- ○ Only if the patient or his partner prefers to know
- ○ No

Dit element tonen

Als Would you communicate about prevention of dementia or cognitive de... No Is niet geselecteerd

If yes, what would you discuss as potential means to reduce the risk of dementia/cognitive decline? I address:

Multiple answers can be selected ptcase4means

- ◻ Diet and nutrition
- ◻ Exercise
- ◻ Keeping the brain active (cognitively stimulating activities)
- ◻ Medication for modifiable risk factors such as hypertension or diabetes
- ◻ Quit smoking
- ◻ More moderate alcohol consumption
- ◻ Reduce stress
- ◻ Sleep
- ◻ Maintaining social contacts and relationships
- ◻ All of the above
- ◻ Other, namely:_____

Dit element tonen

Als Would you communicate about prevention of dementia or cognitive de... No Is niet geselecteerd

Would you personalize this preventive strategy? ptcase4personalize

- ○ No
- ○ Yes

Dit element tonen

Als	Would you personalize this preventive strategy?	Yes	Is geselecteerd

If yes, based on what?
Multiple answers can be selected ptcase4personalizeyes

- ◻ Demographics
- ◻ Diagnostic test results (e.g. cognitive tests, biomarkers)
- ◻ Anamnestic information (e.g., what they have told about lifestyle)

Dit element tonen

Als	Would you communicate about prevention of dementia or cognitive de...	No	Is geselecteerd

Why not?
Multiple answers can be selected ptcase4preventionno

- ◻ I don’t know enough about this topic
- ◻ This is not relevant for this patient
- ◻ I don’t think my patients are interested in this type of information
- ◻ It is difficult to talk about this without accusing/putting the blame on the patient
- ◻ This topic is of lower priority and usually there is no time to discuss this during the consultation
- ◻ Too speculative/there is not yet enough evidence about effective prevention strategies
- ◻ There are no guidelines which I can follow
- ◻ Other, namely:_____

____

Consider the previous patient, but now cerebrospinal fluid measures amyloid-beta 42, total tau and phosphorylated tau are abnormal.

(A 59 year old woman was referred to the memory clinic at her own request by her general practitioner. She experiences memory problems, such as forgetting her keys. She feels a lot of anxiety at these moments. She works part-time as a social worker. MMSE is 29/30. The neuropsychological test scores are all within limits.)

Would you communicate Subjective Cognitive Decline (SCD) as the patient’s syndrome diagnosis/diagnostic label? ptcase5diagnosis

- ○ Yes

- Yes, but I would only communicate a syndrome diagnosis if the patient prefers to know
- No, I would not communicate any syndrome diagnosis or any diagnostic label (I would summarize test results in my own words)
- No, I would communicate another syndrome diagnosis/diagnostic label, namely:_____

Would you communicate the biomarker results to this patient?

ptcase5biomarker

- Yes, I would communicate the biomarker results, yet emphasize that we do not know exactly what this means for the patient
- Yes, I would communicate that the biomarker results imply the presence of Alzheimer's disease
- No, I would not communicate the biomarker results

Dit element tonen

Als Would you communicate the biomarker results to this patient? No, I would not communicate the biomarker results Is geselecteerd

If not, why? I would not disclose because…
Multiple answers can be selected ptcase5biomarkerno

- ☐ It is not included in national care guidelines
- ☐ Disclosure may upset/worry the patient (and/or (care) partner)
- ☐ The information is too difficult for the patient to understand
- ☐ I am not sure how to interpret the results myself
- ☐ There is no added value over a clinical diagnosis
- ☐ There are no implications for the prognosis of an individual patient
- ☐ I feel not equipped to disclose these results
- ☐ Other, namely:_____

Would you communicate prognosis in terms of dementia risk to this patient? ptcase5prognosis

- ○ Yes
- ○ Only if the patient or his partner prefers to know
- ○ No

Dit element tonen

Als Would you communicate prognosis in terms of dementia risk to this ... No Is niet geselecteerd

If so, would you personalize this risk? ptcase5personalizerisk

- ○ No
- ○ Yes

Dit element tonen

Als If so, would you personalize this risk? Yes Is geselecteerd

If yes, based on what?
Multiple answers can be selected ptcase5personalizeriskyes

- ☐ Demographics
- ☐ Diagnostic test results (i.e., in this case, positive biomarkers indicate a higher risk of developing dementia due to Alzheimer's disease)
- ☐ Anamnestic information
- ☐ My experience

Would you communicate about the following aspects of post diagnostic-support with this patient <u>during the disclosure consultation</u>, such as:

ptcase5support

	Yes	No
Counselling by a specialized nurse?	○	○
Peer support groups?	○	○
Disease specific organizations, e.g. Alzheimer's associations?	○	○
Clinical trial opportunities?	○	○
Information on driving?	○	○
Advanced care planning?	○	○
Case management?	○	○
Informational websites?	○	○

Would you communicate about prevention of dementia or cognitive decline with this patient?

ptcase5prevention

- ○ Yes
- ○ Only if the patient or his partner prefers to know
- ○ No

Dit element tonen

Als Would you communicate about prevention of dementia or cognitive de... No Is niet geselecteerd

If yes, what would you discuss as potential means to reduce the risk of dementia/cognitive decline? I address:

Multiple answers can be selected ptcase5means

- ◻ Diet and nutrition
- ◻ Exercise
- ◻ Keeping the brain active (cognitively stimulating activities)
- ◻ Medication for modifiable risk factors such as hypertension or diabetes
- ◻ Quit smoking
- ◻ More moderate alcohol consumption
- ◻ Reduce stress
- ◻ Sleep
- ◻ Maintaining social contacts and relationships
- ◻ All of the above
- ◻ Other, namely:_____

Dit element tonen

Als Would you communicate about prevention of dementia or cognitive de... No Is niet geselecteerd

Would you personalize this preventive strategy? ptcase5personalize

- ○ No
- ○ Yes

Dit element tonen

Als Would you personalize this preventive strategy? Yes Is geselecteerd

If yes, based on what?

Multiple answers can be selected ptcase5personalizeyes

- ◻ Demographics
- ◻ Diagnostic test results (e.g. cognitive tests, biomarkers)
- ◻ Anamnestic information (e.g., what they have told about lifestyle)

Dit element tonen

Als	Would you communicate about prevention of dementia or cognitive de...	No	Is geselecteerd

Why not?
Multiple answers can be selected ptcase5preventionno

- ◻ I don’t know enough about this topic
- ◻ This is not relevant for this patient
- ◻ I don’t think my patients are interested in this type of information
- ◻ It is difficult to talk about this without accusing/putting the blame on the patient
- ◻ This topic is of lower priority and usually there is no time to discuss this during the consultation
- ◻ Too speculative/there is not yet enough evidence about effective prevention strategies
- ◻ There are no guidelines which I can follow
- ◻ Other, namely:_____

Dit element tonen

Als What is your profession? Physician, after completion of specialist training Is geselecteerd

Of What is your profession? Physician, currently in specialist training Is geselecteerd

Of What is your profession? Physician, not in/without specialist training Is geselecteerd

Aducanumab

Disease-modifying treatments

The US Food and Drug Administration (FDA) has recently granted conditional approval of Aducanumab for the treatment of patients with mild cognitive impairment or mild dementia due to Alzheimer's disease. The drug Aducanumab reduces amyloid plaque load in the brain. The EMA (European Medicines Agency) is currently examining the application for approval in Europe.

In case the European Medicines Agency (EMA) would (conditionally) approve Aducanumab, would you consider prescribing it? aducanumab

- Yes
- No
- I don't know

Please explain your answer: aducanumabexplanation

____

For each statement please indicate how much you disagree or agree.

After a (conditional) approval of Aducanumab by the European Medicines Agency (EMA), I will be more likely to:

aducanumabstatements

	1= Strongly disagree	2 = Disagree	3 = Neither agree nor disagree	4 = Agree	5 = Strongly agree
pursue AD biomarker testing in patients without dementia (e.g. SCD and/or MCI)?	○	○	○	○	○
communicate amyloid status to patients without dementia (e.g. SCD and/or MCI)?	○	○	○	○	○
communicate about prognosis with patients without dementia (e.g. SCD and/or MCI)?	○	○	○	○	○
communicate about prevention with patients without dementia (e.g. SCD and/or MCI)?	○	○	○	○	○

Dit element tonen

Als This survey is aimed at clinicians (e.g., physicians, nurses, psyc... with regard to the diagnostic work up for dementia — No, I do not have clinical experience Is niet geselecteerd

Part 4. Clinician-patient communication– additional topics

Part 4 - Clinician-patient communication– additional topics

On the following pages we provide you with some additional questions regarding your opinion on: 1) where stimulation of prevention efforts should take place, 2) using and receiving communication support/tools, 3) your preferred role in decision making, and 4) dealing with uncertainty.

In your view, who's most responsible for discussing prevention with regard to cognitive decline/brain health with patients? *Please rank each of the following answers in order of your preference with #1 being most responsible* preventioneffortsplace

The general practitioner/primary care physician	_____
The referring medical doctor	_____
The memory clinic nurse	_____
The memory clinic treating medical doctor	_____
Public health initiatives (e.g. government campaigns)	_____

Have you participated in any communication skills training/courses? communicationcourse

- o No
- o Yes, as part of my education
- o Yes, after completing my education
- o Yes, both during my education as after completion of my education

Dit element tonen

Als Have you participated in any communication skills training/courses... No Is niet geselecteerd

How long ago was the most recent communication course that you participated in? timecommunicationcourse

- This month
- This year
- More than a year ago
- More than five years ago
- More than ten years ago

If there were online communication tools and/or communication skills training programs available that offered support to clinicians in communicating with patients, for example about the purpose of testing, test results, diagnosis, prognosis or support, would you like to use them? usecommunication

- Yes, I would use the communication tools
- Yes, I would take part in the communication skills training
- Yes, I would use both the communication tools and take part in the skills training
- No
- I don’t know

For which communication skills would you like to receive support, by means of a tool or training? For each skill, please indicate the amount of support you would like to receive on a scale from 1-5, 1 = very little 5 = very much

Skills on how to:

communicationskills

	1 = very little	2	3	4	5 = very much	N/A
involve patients and care partners in decision making, e.g., about diagnostic testing	○	○	○	○	○	○
identify issues the patient wishes to address during the conversation	○	○	○	○	○	○
actively involve both patient and their care partner in the conversation (to manage triadic interactions)	○	○	○	○	○	○
to show empathy (acknowledge and respond to the patient’s views and feelings)	○	○	○	○	○	○
communicate Mild Cognitive Impairment as a diagnosis/diagnostic label	○	○	○	○	○	○
communicate about the dementia risk	○	○	○	○	○	○
communicate uncertainty	○	○	○	○	○	○
stimulate/ensure patient’s understanding of the provided information	○	○	○	○	○	○
communicate with patients who do not (fluently) speak my native language	○	○	○	○	○	○
optimize remote/online consultations	○	○	○	○	○	○

Dit element tonen

Als What is your profession? Physician, after completion of specialist training Is geselecteerd

Of What is your profession? Physician, currently in specialist training Is geselecteerd

Of What is your profession? Physician, not in/without specialist training Is geselecteerd

What is your preferred role in decision making about AD biomarker testing?

preferredrole

- ○ The patient makes the final decision about whether or not to pursue diagnostic testing and which tests to use.
- ○ The patient makes the final decision about whether or not to pursue diagnostic testing and which tests to use after seriously considering the clinician's/my opinion.
- ○ The patient and the clinician/I share the responsibility for deciding about whether or not to pursue diagnostic testing and which tests are best for the patient.
- ○ The clinician makes/I make the final decision about whether or not to pursue diagnostic testing and which tests to use, but seriously consider the patient’s opinion.

- The clinician makes/I make the final decision about whether or not to pursue diagnostic testing and which tests to use.

Listed below are statements related to health care professionals' ability with coping with uncertainty. For each statement please indicate how much you disagree or agree. uncertainty

	1= strongly disagree	2= moderately disagree	3= slightly disagree	4= slightly agree	5= moderately agree	6= strongly agree
I usually feel anxious when I am not sure of a diagnosis	○	○	○	○	○	○
I find the uncertainty involved in patient care disconcerting	○	○	○	○	○	○
Uncertainty in patient care makes me uneasy	○	○	○	○	○	○
I am quite comfortable with uncertainty in patient care	○	○	○	○	○	○
The uncertainty in patient care often troubles me	○	○	○	○	○	○
When I am uncertain of a diagnosis, I imagine all sorts of bad scenarios – patient dies, patient sues, etc…	○	○	○	○	○	○
I fear being held accountable for the limits of my knowledge	○	○	○	○	○	○
I worry about malpractice when I do not know a patient's diagnosis	○	○	○	○	○	○
When clinicians are uncertain of a diagnosis, they should share this information with their patients	○	○	○	○	○	○
I always share my uncertainty with my patients	○	○	○	○	○	○
If I shared all of my uncertainties with my patients, they would lose confidence in me	○	○	○	○	○	○
Sharing my uncertainty improves my relationship with my patients	○	○	○	○	○	○

Lastly, we provide you with statements on which you can provide your opinion on a scale ranging from 'strongly disagree' to 'strongly agree'. statements

	1 = Strongly disagree	2 = Disagree	3 = Neither agree nor disagree	4 = Agree	5 = Strongly agree
As a clinician, it is part of my job to help patients cope with their situation/symptoms.	○	○	○	○	○
It is important to provide emotional support to patients and their care partners.	○	○	○	○	○

It is important to discuss from the start of the diagnostic trajectory what patients want to know and what they don’t want to know.	○	○	○	○	○
Patients’ needs and preferences should be leading in the provision of diagnostic care.	○	○	○	○	○
Too much information will lead to an information overload and thus an unhappy patient, so it is better to not tell the patient everything.	○	○	○	○	○
We should not (yet) inform individuals with Subjective Cognitive Decline about their Alzheimer’s disease biomarker status.	○	○	○	○	○
We should not (yet) inform patients with Mild Cognitive Impairment about their Alzheimer’s disease biomarker status.	○	○	○	○	○
We should communicate a personalized prognosis to patients who do not have dementia (yet), i.e., tailoring dementia risk information based on characteristics such as age and biomarker status.	○	○	○	○	○
It is important to explain the difference between dementia and Alzheimer’s disease to patients.	○	○	○	○	○
Considering Alzheimer’s disease as a biological construct is part of a research framework and not yet suitable for clinical practice.	○	○	○	○	○
Prevention efforts should be part of memory clinic care.	○	○	○	○	○

Thank you, your answers were saved perfectly!

Would you like to be involved in future studies or informed about the survey results in the context of EU-FINGERS and LETHE projects?

Please click here. This will take you to a short form where you can fill in your contact details, so that these personal data will be stored separate from your survey answers.
