# Supplementary material for: Communication about diagnosis, prognosis, and prevention in the memory clinic: perspectives of European memory clinic professionals
Source: Alzheimers Res Ther. 2023 Aug 5;15:131. doi: 10.1186/s13195-023-01276-9 (PMC10404377; doi:10.1186/s13195-023-01276-9)
Supplement: Supplementary file 2 — Additional file 2: Supplementary Table 1. Patient cases: communicating about diagnosis and diagnostic tests. Supplementary Table 2. Patient cases: communicating about prognosis and prevention. [file 13195_2023_1276_MOESM2_ESM.docx]

**Supplementary Material**

**Table 1**. Patient cases: communicating about diagnosis and diagnostic tests

|  | **Abnormal AD biomarkers** | | | **Normal AD biomarkers** | |
| --- | --- | --- | --- | --- | --- |
|  | **AD dementia** | **MCI +** | **SCD +** | **MCI -** | **SCD -** |
| **Would you communicate the patient’s syndrome diagnosis?** | | | | | |
| No, I would not communicate any syndrome diagnosis or any diagnostic label (I would summarize test results in my own words) | 5 (3%) | 4 (3%) | 37 (23%) | 29 (18%) | 40 (25%) |
| No, I would communicate another syndrome diagnosis/label | 13 (8%) | 15 (9%) | 18 (11%) | 9 (6%) | 5 (3%) |
| Yes sum | 142 (88%) | 141 (88%) | 105 (66%) | 122 (77%) | 115 (72%) |
| Yes | 119 (74%) | 126 (79%) | 85 (53%) | 102 (64%) | 103 (64%) |
| Yes, but I would only communicate a syndrome diagnosis if the patient prefers to know | 23 (14%) | 15 (9%) | 20 (13%) | 20 (13%) | 12 (8%) |
| **Would you communicate Alzheimer’s disease as the underlying pathology to this patient?** | | | | | |
| (Yes/no; nr answered ‘yes’) | 155 (97%) | n/a | n/a | n/a | n/a |
| **Would you explain the difference between dementia and Alzheimer’s disease?** | | | | | |
| (Yes/no; nr answered ‘yes’) | 147 (92%) | n/a | n/a | n/a | n/a |
| **Would you communicate the biomarker results to this patient?** | | | | | |
| No, I would not communicate the biomarker results | n/a | 1 (1%) | 5 (3%) | 0 | 3 (2%) |
| Yes sum |  | 159 (100%) | 155 (97%) | 160 (100%) | 157 (98%) |
| Yes, I would communicate the biomarker results, yet emphasize that we do not know exactly what this means for the patient | n/a | 51 (32%) | 109 (68%) | 68 (43%) | 47 (29%) |
| Yes, I would communicate the biomarker results and tell that the patient currently does not have AD / Yes, I would communicate that the biomarker results imply the presence of AD | n/a | 108 (68%) | 46 (29%) | 92 (58%) | 110 (69%) |

Data are presented as n (%).

**Table 2.** Patient cases: communicating about prognosis and prevention

|  | **Abnormal AD biomarkers** | | | **Normal AD biomarkers** | |
| --- | --- | --- | --- | --- | --- |
|  | **AD dementia** | **MCI +** | **SCD +** | **MCI -** | **SCD -** |
| **Would you communicate about prognosis to this patient?** | | | | | |
| No | 6 (4%) | 4 (3%) | 17 (11%) | 17 (11%) | 33 (21%) |
| Yes sum | 154 (97%) | 156 (98%) | 143 (89%) | 143 (89%) | 127 (79%) |
| Yes | 100 (63%) | 124 (78%) | 85 (53%) | 97 (61%) | 85 (53%) |
| Only if the patient or partner prefers to know | 54 (34%) | 32 (20%) | 58 (36%) | 46 (29%) | 42 (26%) |
| **If so, would you personalize the prognosis?** | | | | | |
| No | n/a | n/a | 31 (19%) | n/a | 43 (27%) |
| No, I would just explain that individuals with MCI have a higher chance of developing dementia | n/a | 43 (27%) | n/a | 60 (38%) | n/a |
| No, I would explain the chance of developing dementia is fifty-fifty, in this case | n/a | 9 (6%) | n/a | 7 (4%) | n/a |
| Yes | n/a | 104 (65%) | 112 (70%) | 76 (48%) | 84 (53%) |
| **If yes, based on what?** (Multiple answers could be selected) | | | | | |
| Diagnostic test results (i.e., negative biomarkers indicate a low risk of developing dementia due to AD / positive biomarkers indicate a high(er) risk of developing dementia due to AD) | n/a | 100 (63%) | 108 (68%) | 70 (44%) | 81 (51%) |
| Anamnestic information | n/a | 66 (41%) | 86 (54%) | 53 (33%) | 67 (42%) |
| My experience | n/a | 49 (31%) | 57 (36%) | 41 (26%) | 41 (26%) |
| Demographics | n/a | 41 (26%) | 54 (34%) | 33 (21%) | 40 (25%) |
| **Would you communicate about prevention to this patient?** | | | | | |
| No | 5 (3%) | 2 (1%) | 6 (4%) | 8 (5%) | 16 (10%) |
| Yes sum | 155 (97%) | 158 (99%) | 154 (97%) | 152 (95%) | 144 (90%) |
| Yes | 122 (76%) | 144 (90%) | 124 (78%) | 119 (74%) | 104 (65%) |
| Only if the patient or partner prefers to know | 33 (21%) | 14 (9%) | 30 (19%) | 33 (21%) | 40 (25%) |
| **Would you personalize the preventive strategy?** | | | | | |
| (Yes/no; nr answered ‘yes’) | 131 (82%) | 137 (86%) | 128 (80%) | 132 (83%) | 117 (73%) |
| **If yes, based on what?** (Multiple answers could be selected) | | | | | |
| Anamnestic information (e.g., what they have told about lifestyle) | 123 (77%) | 131 (82%) | 124 (78%) | 126 (79%) | 114 (71%) |
| Diagnostic test results (e.g. cognitive tests, biomarkers) | 63 (39%) | 92 (58%) | 88 (55%) | 76 (48%) | 71 (44%) |
| Demographics | 37 (23%) | 56 (35%) | 57 (36%) | 47 (29%) | 48 (30%) |

Data are presented as n (%).
